# A Resource-Efficient Hybrid Deep Learning Framework with External Validation for Intracranial Hemorrhage Detection in CT Scans

**DOI:** 10.3390/diagnostics16172776

**Published:** 2026-08-29

**Authors:** José Rafael Peña Gutiérrez, César Julio Bustacara Medina

**Affiliations:** Department of Systems Engineering, Pontificia Universidad Javeriana, Bogotá 110231, Colombia; jrafael_pena@javeriana.edu.co

**Keywords:** intracranial hemorrhage, computed tomography, deep learning, cross-domain generalization, clinical decision support, computational efficiency, medical image analysis

## Abstract

**Background:** Intracranial hemorrhage (ICH) is a time-critical neurological emergency in which delayed diagnosis significantly worsens patient outcomes. This challenge is amplified in resource-limited, high-workload settings where rapid neuroimaging interpretation may be constrained. Many high-performing deep learning approaches for disease detection rely on large-scale models trained on extensive data and evaluated only on internal datasets, limiting their generalization across heterogeneous clinical environments. This study aims to develop and evaluate a resource-efficient framework for automated ICH detection from CT scans, with internal and external validation across heterogeneous clinical settings. **Methods:** A hybrid deep learning framework was developed, combining an EfficientNetV2-S-based feature extractor with a bidirectional GRU model for scan-level prediction. The model was trained on a stratified subset of 6000 CT scans from the RSNA Intracranial Hemorrhage Detection dataset and evaluated using an internal test set and two external validation cohorts (PhysioNet and CQ500). **Results:** On an internal held-out test set, the model achieved scan-level AUROC and AUPRC of 0.980 and 0.977, respectively, and slice-level AUROC and AUPRC of 0.981 and 0.923. External validation on the PhysioNet and CQ500 datasets yielded scan-level AUROC/AUPRC values of 0.914/0.932 and 0.905/0.909, respectively, demonstrating consistent performance across datasets differing in institution, geography, patient population, and acquisition protocols. **Conclusions:** Despite its compact architecture and reduced training subset, the proposed framework achieves performance competitive with substantially larger and more computationally demanding models, completing training in under 26 h on single-GPU hardware. These results support the feasibility of reproducible, resource-efficient ICH detection systems for automated triage in emergency radiology workflows across different clinical settings.

## 1. Introduction

Intracranial hemorrhage (ICH), defined as bleeding within the cranial cavity, is a neurological emergency associated with high morbidity and mortality [1,2]. According to data from the Global Burden of Disease 2021 study, approximately 3.4 million deaths worldwide were attributed to ICH, reflecting a 41.29% increase between 1990 and 2021 [3]. Considering that nearly half of ICH-related deaths occur within the initial 24 h, early detection and rapid intervention are crucial [4].

Neuroimaging is essential for achieving a timely diagnosis. While both computed tomography (CT) and magnetic resonance imaging (MRI) are commonly used in clinical practice, CT is preferred for its speed and availability [2,4]. Traditionally, CT scans are interpreted by radiologists and other medical specialists, but the growing volume of cases has strained radiology workflows, potentially delaying diagnosis and treatment [5]. This challenge is intensified in low-income countries, where healthcare resources are more limited and the incidence of ICH is approximately twice as high as in high-income regions [4].

In settings with limited medical resources, automated detection systems can serve as effective decision support tools, helping radiologists manage high imaging volumes [6,7]. During peak periods in emergency departments, these systems can prioritize CT scans based on the estimated probability of ICH, bringing the most critical cases to attention first rather than strictly following arrival order [6,8]. This triage approach is especially helpful when specialist availability is limited [5] and trauma cases are frequent, while preserving the clinician’s final diagnostic authority [5,7]. Overall, these systems aim to provide reliable confidence estimates that support clinical decision-making, improve workflow prioritization, and reduce time to diagnosis in time-critical settings.

In this context, artificial intelligence (AI)-based systems, particularly those leveraging deep learning (DL), offer promising tools to support radiological workflows by providing automated and precise detection of pathologies [9,10]. DL has already demonstrated remarkable success across various medical imaging tasks, in some cases reaching a level comparable to that of trained professionals [11,12].

Within research on ICH, DL has increasingly been applied to detection and characterization tasks [13], primarily focusing on two main objectives: classification—to determine the presence of hemorrhage and/or identify its specific subtype—[14,15,16,17] and segmentation to delineate the hemorrhagic regions within CT scans [18,19,20].

For classification tasks, many studies employ 2D convolutional neural networks (CNNs) to extract feature maps from individual CT slices and perform slice-level prediction and model evaluation [14,15,21,22,23]. While this approach is effective for detecting localized hemorrhagic patterns, it treats each slice independently and does not explicitly model the spatial relationships between consecutive slices within a scan. To capture these inter-slice dependencies and produce scan-level predictions, several studies have extended this framework by integrating recurrent neural networks (RNNs) that sequentially process slice-level features [16,24]. More recently, advances in attention-based architectures have also been adopted in this domain, with several studies exploring Vision Transformers (ViTs) and attention-driven sequential models as alternatives to CNN–RNN pipelines [25,26].

Adoption, exploration, and reproducibility of these models in real-world clinical environments can face barriers such as methodological transparency, dataset availability, and computational requirements [27,28]. For ICH detection models, many of the highest-performing approaches rely either on large-scale architectures or on ensemble methods [14,24,29], trained on extensive datasets, resulting in substantial computational costs during both model development and training. Most of these applications rely on large backbones, ensemble configurations, or training on substantially larger datasets, resulting in higher computational demands during model development and experimentation.

In addition to computational constraints, another important challenge is model generalization [30]. Despite strong performance reported on internal datasets, comprehensive external validation using clinical data from different institutions and geographic regions remains limited in the literature. These observations highlight the need for approaches that balance predictive performance with computational efficiency while maintaining robustness across diverse clinical environments. To address these challenges, this study proposes a resource-efficient hybrid deep learning framework for automated ICH detection from CT scans. The framework is trained on a stratified subset of the publicly available Radiological Society of North America (RSNA) Intracranial Hemorrhage Detection dataset and evaluated for generalization on two independent external cohorts: the PhysioNet dataset, which differs from RSNA in institution, geographic origin, and patient population, and the CQ500 dataset, which further challenges generalization through substantial differences in acquisition protocol and slice thickness. Unlike many high-performing approaches that rely on large-scale architectures, ensemble configurations, or full-dataset training, the proposed framework combines resource-efficient slice-level feature extraction with inter-slice sequential modeling and explicit scan-level supervision within a compact architecture trainable on consumer-grade hardware. By pairing this design with evaluation on two independent external cohorts, the study seeks to address both the reproducibility and generalizability gaps that persist in the current ICH detection literature.

## 2. Literature Review

### 2.1. Preprocessing

Preprocessing techniques are generally less emphasized in DL frameworks, as these architectures can automatically learn relevant patterns from the rich feature representations present in medical images. However, image windowing remains one of the most widely adopted preprocessing strategies in CT-based disease detection. CT images are represented in Hounsfield Units (HU)—a standardized measure of radiodensity ranging approximately from −1024 to +3071 [31].

The wide dynamic range of HU values makes it difficult to visualize subtle intensity variations across an entire CT slice. Moreover, most convolutional neural network (CNN) architectures were originally designed for natural images with an 8-bit intensity range (0–255), implicitly assuming a limited dynamic spectrum. Windowing addresses this issue by selecting a clinically relevant portion of the HU range and mapping it into a narrower intensity interval. This process is controlled by two parameters: the window center (WC), which defines the midpoint HU value of interest, and the window width (WW), which determines the range of HU values displayed around that center.

In many studies, a single window setting is applied to each slice before it is used as input to the network. However, several works have adopted a multi-window strategy to better capture tissue-specific information. In this approach, multiple windowed versions of the same slice—each using different WC/WW settings—are generated and stacked as a multi-channel input [32]. This strategy not only highlights complementary anatomical structures but also aligns naturally with the three-channel input format expected by many CNN architectures.

The most frequently used window settings are summarized in Table 1.

For instance, Lee et al. [14] reported that employing multi-window conversions significantly improved model performance, while Wang et al. [33] found that using three windows (brain, subdural, and bone), combined with a sigmoid-based intensity mapping (instead of standard hard clipping), further enhanced results compared to using a single window. In a different approach, Hyunkwang et al. [36] proposed a deeper optimization of the windowing strategy by introducing a trainable windowing layer, which—together with a sigmoid function—learns the optimal settings for mapping the raw HU values.

Other preprocessing techniques reported in the literature include skull and face removal from CT images using thresholding and morphological operations, aimed at retaining only intracranial structures to enhance privacy and reduce task complexity [15]. In another approach, region-growing algorithms were applied to extract potential ICH lesion areas from unsupervised segmentation results, guided by physician-defined gray value ranges across three window settings. These segmentation maps were subsequently refined through Gaussian smoothing and merged to generate weak segmentation labels [35]. Furthermore, contrast enhancement techniques such as CLAHE have been employed to improve image quality by limiting local contrast amplification and applying histogram equalization with block-wise interpolation, thereby reducing noise and improving performance in detection and segmentation tasks [21].

### 2.2. Label Granularity and Training Strategies

An important consideration in ICH detection frameworks is the level of label granularity used during model training. Labels may be provided at the slice level—indicating the presence of hemorrhage in individual CT slices—or at the scan (study) level, where the label reflects whether any slice within the scan contains hemorrhage. Slice-level labels provide more detailed supervision and allow models to learn localized pathological patterns, but they are costly to obtain and require extensive annotation effort from neuroradiologists [16,34]. In contrast, scan-level labels are easier to acquire because they only indicate the presence of hemorrhage within the entire examination.

Several studies have explored the impact of supervision granularity on model performance. Ye et al. [16], for example, compared models trained with slice-level and scan-level annotations using a scan-level evaluation. While both approaches achieved strong performance in binary hemorrhage detection, the model trained with slice-level supervision showed improved performance in the multi-class task of identifying ICH subtypes, highlighting the benefits of localized information for fine-grained classification.

To address the limited availability of detailed slice-level annotations, other works have explored multiple instance learning (MIL), a framework that enables models to infer instance-level (slice) predictions from bag-level (scan) labels [34,37]. In this paradigm, an entire CT scan is treated as a bag of slices, and the model learns to associate slice-level patterns with the overall scan-level label. However, MIL approaches are inherently constrained by the weak supervision signal: without explicit slice-level labels, the model must infer localized hemorrhage patterns solely from scan-level feedback, which can limit the precision of slice-level predictions and complicate optimization in deeply imbalanced datasets where positive slices constitute a small fraction of each bag.

Overall, these approaches highlight the trade-off between detailed slice-level supervision and the broader contextual information available at the scan level, motivating architectures capable of leveraging both local and global information when analyzing volumetric CT studies.

### 2.3. Model Architectures

For the processing stage, a common approach involves using CNN architectures to extract feature maps from individual slices [13,32]. These architectures are typically large and trained on millions of images [38,39], requiring significant computational resources. Given that annotated data for ICH classification is often limited and computing power may be constrained, transfer learning (TL) and fine-tuning are widely adopted.

Instead of training CNNs from scratch with randomly initialized weights, many studies leverage pre-trained CNN models—originally trained on large-scale datasets—and fine-tune them using ICH-specific datasets [15,16,22,24,29,33,37]. This approach allows researchers to benefit from the rich feature representations learned from general-purpose images while adapting the models to the nuances of medical imaging data.

However, 2D-CNN architectures are inherently limited in their ability to capture inter-slice context and the sequential dependencies present in volumetric data such as CT scans. Several studies have reported performance improvements or enhanced robustness by incorporating methods that model these spatial–temporal relationships, in contrast to approaches that rely exclusively on intra-slice features extracted by 2D-CNNs [13,16,24,32].

One common approach to modeling sequential dependencies and capturing inter-slice information is the integration of recurrent neural network (RNN) architectures, which typically receive a flattened version of the slice-level feature maps as input [16,24,40,41,42]. For example, Ye et al. [16] trained a joint CNN-RNN model on a dataset of 2836 scans, a convolutional architecture with approximately 138 million parameters [43], as the CNN backbone to extract features from individual slices. These features were then passed to a two-layer bidirectional Gated Recurrent Unit (GRU) network with 32-dimensional hidden states, enabling the model to capture sequential patterns across slices before producing final classification probabilities.

3D-CNN architectures are also used, as they inherently process volumetric data such as CT scans [35,44]. Unlike 2D CNNs, these models operate on the full volume of slices, eliminating the need for hybrid CNN-RNN architectures. However, systematic reviews have identified several drawbacks that limit the effectiveness of 3D CNNs. By design, they handle an additional spatial dimension, which introduces computational challenges such as increased memory usage and parameter counts, restricting the feasible depth and capacity of the architecture due to hardware constraints. Moreover, the curse of dimensionality demands exponentially larger labeled datasets for effective training, increasing the risk of overfitting when data is scarce. Consequently, multiple studies have reported that 2D CNNs and CNN-RNN hybrid models often outperform 3D CNNs in tasks like ICH detection [13,32].

Recently, with the introduction of Transformer architectures and self-attention mechanisms, several studies have begun to replace conventional CNN-based architectures with Vision Transformers (ViTs) and attention-based models [23,29,33,42]. For instance, Hossain et al. [42] replaced the CNN backbone with a ViT architecture to obtain feature maps for each slice, which were then sequentially processed using a GRU network. Lezzar et al. [29] proposed a multi-head approach in which Transformer blocks were used to refine the feature maps produced by the CNN backbone—incorporating skip connections and ViT-style patch processing. These refined features were subsequently aggregated using a hierarchical attention mechanism to capture both intra-slice and inter-slice dependencies. Similarly, Wang et al. [33], Wu et al. [34], and Kumar Kar et al. [23] retained a CNN backbone for intra-slice feature extraction but replaced RNN components with Transformer-based attention mechanisms to model inter-slice relationships more effectively.

While recent advances in ICH detection have achieved strong performance through large-scale architectures [16,24,29,33,35] and extensive training datasets [22,24,33,35], these approaches often require substantial computational resources that may not be readily available in many clinical environments. In addition, many studies either focus exclusively on slice-level classification or derive scan-level predictions through post-hoc aggregation, making it difficult to jointly optimize clinically relevant study-level decisions while retaining localized supervision. External validation also remains limited across much of the literature, particularly across cohorts that simultaneously introduce demographic, institutional, and technical distribution shifts.

Motivated by these challenges, this study proposes a hybrid deep learning framework designed to balance predictive performance with computational efficiency. The proposed approach combines a compact CNN backbone for efficient slice-level feature extraction with a bidirectional GRU network to capture sequential dependencies across slices within a CT scan. To generate scan-level predictions, the sequential representations are aggregated using complementary pooling strategies, including attention-based weighting that enables the model to emphasize diagnostically relevant slices. This design allows the framework to leverage both local slice-level features and global scan-level context while maintaining a resource-efficient architecture suitable for reproducible development and deployment across diverse clinical settings.

## 3. Materials and Methods

### 3.1. Datasets

#### 3.1.1. RSNA ICH Dataset

This study employs the 2019 RSNA ICH Detection Challenge dataset [45] for model training. The dataset comprises 27,861 cranial CT scans and a total of 1,074,271 slices, making it one of the largest publicly available datasets for ICH detection. The data were collected from three institutions: Stanford University, Universidade Federal de São Paulo, and Thomas Jefferson University [45].

The RSNA dataset was selected because it combines several characteristics that are particularly valuable for developing and evaluating sequence-based ICH detection models. It is one of the largest publicly available ICH datasets, providing sufficient diversity to investigate resource-efficient training on a stratified subset while maintaining independent validation and test partitions. In addition, the dataset includes expert-annotated slice-level labels required for the dual-level training strategy adopted in this work. Finally, because the data originate from three independent institutions, the dataset contains greater variability in imaging practices and patient populations than many single-center cohorts, supporting the development of models with improved potential for cross-institutional generalization.

Annotations were provided through a collaboration between the Radiological Society of North America (RSNA) and the American Society of Neuroradiology (ASNR). A total of 60 expert annotators, all ASNR members, labeled each CT slice for the presence of one or more ICH subtypes: intraparenchymal (IPH), intraventricular (IVH), subarachnoid (SAH), subdural (SDH), and epidural (EDH). Slices without hemorrhage were also included and labeled as negative for all subtypes [45].

The challenge consisted of two stages, with labels for the stage 2 test set remaining unreleased. Therefore, all supervised learning approaches rely on the stage 2 training set, which comprises 21,744 labeled studies and a total of 752,803 slices. Table 2 summarizes the distribution of labeled studies, where 8882 studies (107,933 slices) are positive for at least one ICH subtype, while 12,862 studies (644,870 slices) contain no hemorrhage. As shown, subdural hemorrhages (SDH) are the most common, present in 6.27% of slices, whereas epidural hemorrhages (EDH) are the least frequent, appearing in only 0.42% of slices.

The images are provided in DICOM (Digital Imaging and Communications in Medicine) format [46]. Each DICOM file contains both the slice array and its associated metadata. Rescaling metadata fields are required to convert raw pixel values into Hounsfield Units (HU), while additional metadata—specifically the StudyInstanceUID and ImagePositionPatient vectors—are used to group slices belonging to the same study (scan). Each CT slice is a 512 × 512 pixel image, which is standard for cranial CT imaging, and the slice thickness ranges from 3 mm to 5 mm. The study-level grouping is essential for sequence-based models, which process slices in their anatomical order within each study. It is also employed when partitioning the dataset into training and test sets to ensure that slices from the same study do not appear in different sets, thereby preventing information leakage.

A subset of the RSNA ICH dataset was constructed to maintain the statistical properties of the complete dataset through stratified sampling at the study level. This sampling strategy preserves both the prevalence rates of each ICH subtype and their co-occurrence patterns.

Although ICH subtypes were used for stratified sampling, this work focuses on ICH detection regardless of subtype. Therefore, the ICH (Any) label is the sole target used for training, making the task a binary classification problem. As shown in Table 3, the final subset comprises 6000 studies (207,592 slices) divided into training, validation, and test sets, with 4400 (152,384), 600 (20,725), and 1000 (34,483) studies, respectively, each preserving the label distribution of the RSNA dataset.

**Figure 1:**
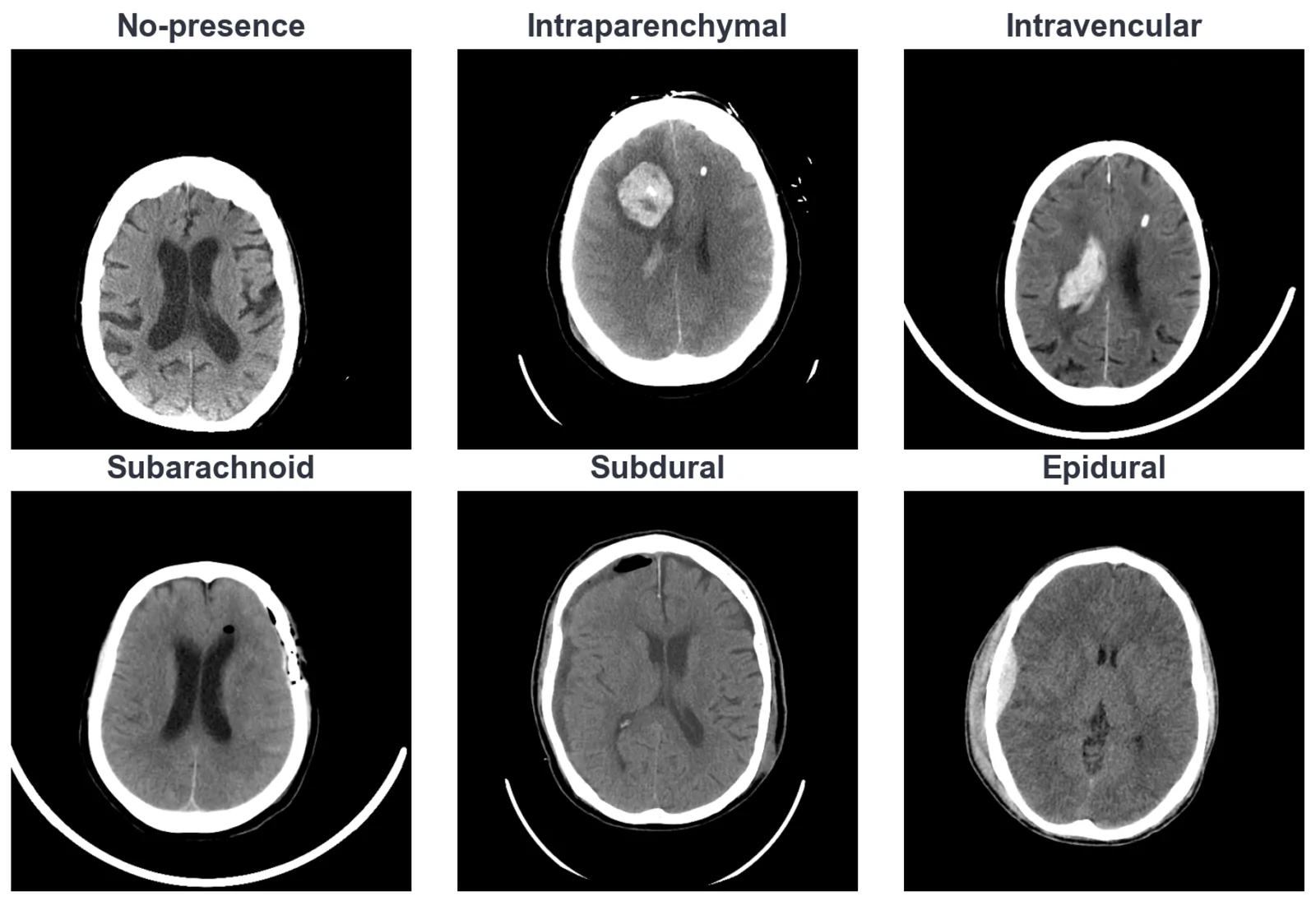
CT slices showing a negative case and the five ICH subtypes: IPH, IVH, SAH, SDH, and EDH.

Figure 1 presents examples of each ICH subtype included in the RSNA dataset, along with a negative case. The slices were obtained from the HU-converted arrays and visualized using the standard windowing parameters specified in the metadata, which are recommended for initial review.

#### 3.1.2. PhysioNet ICH Dataset

To assess the generalization capability of the proposed framework, external validation was performed using the PhysioNet CT Images for ICH Detection and Segmentation dataset [47,48]. This dataset comprises 82 cranial CT scans from patients with traumatic brain injury who were admitted to the emergency unit at Al Hilla Teaching Hospital in Iraq. The scans were collected between February and August 2018, with data acquisition approved by the Iraqi Ministry of Health, Babil Office.

The dataset includes 75 publicly available CT scans in NIfTI format. Each scan contains a mean of 37.2 slices (median: 36 slices) with 5 mm slice thickness. Of the 75 available scans, 36 are positive for ICH and 39 are negative, resulting in a prevalence of 48.0% at the scan level. Each slice was annotated by two radiologists in consensus for the presence of different ICH subtypes (IPH, IVH, SAH, SDH, EDH) as well as skull fractures.

For the purposes of this work, binary ICH labels at both slice and scan levels were utilized, as the PhysioNet dataset serves as an external validation set to evaluate the model’s generalization capability across different institutions, patient populations, imaging acquisition protocols, and geographic regions.

Unlike the RSNA dataset, which includes multi-institutional data from the United States and Brazil, the PhysioNet dataset represents a distinct clinical context: a single-center emergency setting in a developing country with a predominantly younger trauma population. This diversity in data sources provides a rigorous test of the model’s robustness and clinical applicability when deployed in new clinical environments with potentially different demographic and imaging characteristics.

#### 3.1.3. CQ500 Dataset

The CQ500 dataset, curated by Qure.ai and the Centre for Advanced Research in Imaging, Neurosciences and Genomics (CARING) in New Delhi, India, is a publicly available non-contrast head CT dataset released to support the clinical validation of automated ICH detection algorithms [22]. The dataset was collected in two batches (B1 and B2) from multiple radiology centres in New Delhi and comprises 491 studies with a total of 193,317 slices. Each study was independently interpreted by three radiologists with 8, 12, and 20 years of experience in cranial non-contrast CT evaluation, providing scan-level annotations for ICH presence and its subtypes (IPH, IVH, SAH, SDH, EDH), as well as calvarial fractures, midline shift, and mass effect; no single consensus label is provided. Following the original publication, the reference standard was defined as the majority vote across the three radiologist interpretations. No slice-level annotations are available in the original dataset.

The raw release is organized into archive folders containing one or more patient directories, where each patient directory may include multiple DICOM series corresponding to different reconstructions of the same acquisition (e.g., pre-contrast thin-slice, pre-contrast standard, bone kernel, and post-contrast reconstructions). All DICOM files across the dataset were processed and their metadata extracted. Files were first grouped at the study level using patient identifier and study instance UID, which were found to be in strict one-to-one correspondence. Within each study, DICOM files were subsequently grouped by series instance UID to identify candidate reconstruction series. When multiple candidate series were available, non-diagnostic reconstructions were excluded, and the remaining series with the greatest number of slices was retained, which in practice consistently selected the thin-slice pre-contrast reconstruction.

Of the 491 patient identifiers listed in the label file, 473 were successfully matched to DICOM files through the PatientID field and retained for analysis. The remaining 18 patient identifiers could not be matched to any DICOM metadata and no corresponding patient directories were found in the downloaded archives. The retained studies comprised a total of 103,609 slices prior to further preprocessing.

One study (CQ500-CT-163), containing only four slices, was excluded as anatomically insufficient for meaningful inference. Before exclusion, the slice count distribution across the 473 studies ranged from 4 to 1049 slices, with a mean of 219, a median of 246, a 95th percentile of 325, and a 99th percentile of 486. The comparatively high slice counts relative to the RSNA training data, where slice thickness typically ranges from 3 mm to 5 mm, are attributable to the predominance of studies acquired with a slice thickness of 0.625 mm (75%; see Table 4). This difference in acquisition protocol represents a meaningful domain shift relative to the training data and provides a more demanding evaluation of model generalization.

Given the slice count distribution, studies exceeding 500 slices (5 studies) were symmetrically trimmed along the z-axis to preserve the central cranial volume and maintain computational feasibility under GPU memory constraints. Slice ordering was determined using the ImagePositionPatient z-coordinate. After filtering and trimming, the dataset comprised 472 studies and 102,750 slices, with a maximum of 500 slices per study, a mean of 218 slices, and a median of 246. All images had a spatial resolution of 512×512 pixels and were stored in DICOM format. Pixel values were converted to Hounsfield Units using the rescale slope and intercept values provided in the corresponding DICOM metadata.

Scan-level binary ICH labels were derived by majority voting across the three radiologist interpretations provided with the dataset. A study was assigned a positive label when at least two of the three readers identified ICH, consistent with the reference standard criterion used in the original publication [22]. Of the 472 studies, 197 (41.7%) were labeled as positive for ICH and 275 (58.3%) as negative.

Because the CQ500 release does not provide slice-level annotations, slice-level labels were derived from the Brain Hemorrhage Extended (BHX) dataset [49], a publicly available extension of CQ500 hosted on PhysioNet that provides bounding-box annotations for five ICH subtypes at the individual-slice level. BHX annotations were generated by three trained neuroradiologists through extrapolation of thick-slice scan-level interpretations to thin-slice images. A binary slice-level target was assigned to each slice: 1 if its SOP Instance UID appeared among the positive slices in the BHX V3 extrapolation file, and 0 otherwise.

Importantly, BHX annotations do not fully cover the 472 retained studies: some studies are absent from BHX V3, whereas others were annotated only in earlier BHX versions or on series excluded during preprocessing. To assess annotation coverage, a BHX-derived study-level signal was constructed by assigning a positive label to studies containing at least one BHX-positive slice and comparing it with the majority-vote scan-level labels. This comparison revealed 56 disagreements among the original 473 studies: 31 studies labeled as ICH-positive by majority vote but without any BHX-positive slices, and 25 studies labeled as negative by majority vote but containing BHX-positive slices. These discrepancies likely reflect incomplete BHX coverage rather than disagreement between annotation sources.

To ensure consistent slice-level evaluation across all three datasets, slice-level AUC on CQ500 was computed on an agreement subset restricted to studies where the BHX-derived scan-level signal matched the majority-vote label. This subset comprised 416 studies (166 positive and 250 negative), totaling 90,422 slices, of which 11,267 (12.5%) were assigned positive slice-level labels, as summarized in Table 5.

### 3.2. Preprocessing

Images are extracted from DICOM files, converted to Hounsfield Units (HU) through rescaling, and stored as Zarr arrays—an efficient, cloud-native format for large multidimensional data that enables chunked storage and rapid slice-level access during preprocessing [50].

As highlighted in the literature, windowing strategies play a crucial role in ICH detection using CNN-based models. Because most pretrained CNN backbones require three-channel inputs, each strategy in this study produces three windowed images per slice. The following windowing approaches were evaluated:

- Single-window strategy: Applies the brain window (WC = 40, WW = 80) to the HU array and replicates it three times.
- Multi-window strategy: Applies three different windows—brain (WC = 40, WW = 80), subdural (WC = 80, WW = 200), and bone (WC = 400, WW = 1800)—to each HU array.
- Sigmoid multi-window strategy: Applies the same three windows (brain, subdural, and bone) but replaces clipping with a sigmoid-based intensity mapping [33,36].

Additional preprocessing steps included resizing, intensity scaling, and normalization to match the input requirements of the pretrained CNN backbone. After applying the windowing strategy, each slice was resized to (384×384) pixels using bilinear interpolation. The resulting three-channel image was then scaled to a normalized intensity range and standardized using the ImageNet normalization statistics (mean = [0.485, 0.456, 0.406], standard deviation = [0.229, 0.224, 0.225]), consistent with the CNN backbone pretraining procedure.

### 3.3. Data Augmentation

Following preprocessing, data augmentation was employed as a regularization strategy to improve model generalization and reduce overfitting.

Affine transformations are among the most commonly used techniques in ICH-related studies performing slice-level augmentation [16,24,33,42], as well as in CT medical imaging in general [51]. An affine transformation is a geometric mapping that preserves the relative spatial relationships of structures (maintaining straight lines, parallelism, and overall anatomical geometry) while allowing controlled changes in an image’s position, orientation, and/or shape. These transformations preserve anatomical geometry while introducing controlled variability that promotes invariance to non-clinical spatial differences.

**Figure 2:**
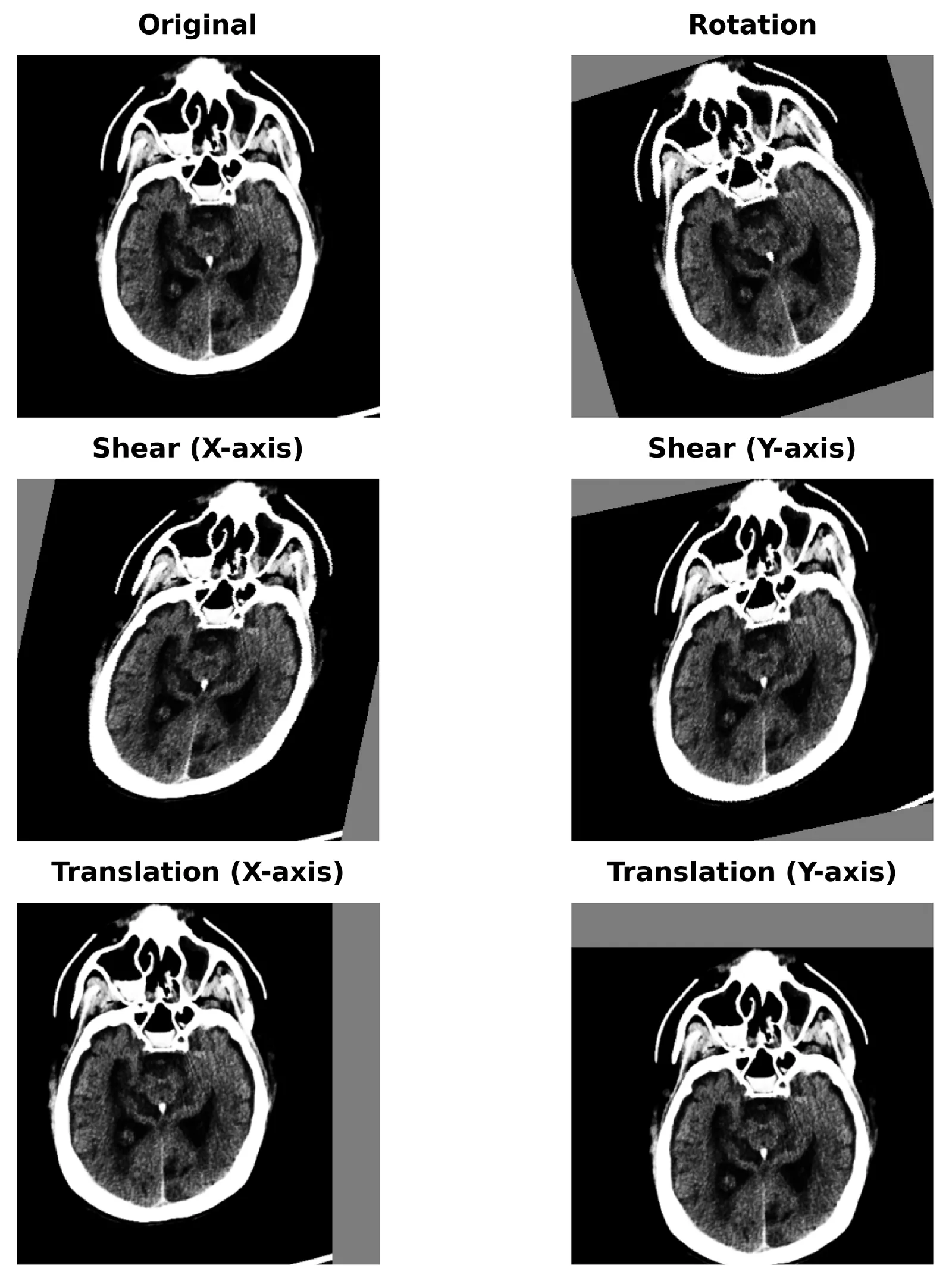
Examples of the spatial augmentations used in this study. From left to right and top to bottom: original slice, rotation, shear (X-axis), shear (Y-axis), translation (X-axis), and translation (Y-axis).

In this study, the applied transformations include vertical flipping, rotation, shearing, and translation. Figure 2 illustrates examples of each spatial augmentation applied to a representative CT slice. While prior studies commonly apply these operations with fixed probabilities, this work adopts the TrivialAugment strategy [52] for cranial CT imaging. TrivialAugment uniformly samples a single transformation and its magnitude per image, introducing diverse yet anatomically plausible perturbations. It also eliminates the need for hyperparameter tuning, reducing search costs while maintaining state-of-the-art performance [53].

A summary of the TrivialAugment implementation used in this study is provided in Table 6.

In addition to spatial operations, pixel-value transformations were considered. Although such methods have been applied in CT imaging [51], they risk altering diagnostically relevant intensity patterns and are therefore used less frequently in ICH detection pipelines. An exception is [33], which applied brightness adjustments and Gaussian noise.

In this study, pixel-value augmentations were incorporated to introduce moderate intensity variability while preserving radiological integrity. These operations follow the same TrivialAugment paradigm: when pixel-value augmentation is triggered, one transform is selected uniformly at random and its parameters are sampled from predefined ranges. Two transformations were implemented: (1) Gaussian noise—adding zero-mean noise with a standard deviation scaled to the sampled magnitude—and (2) Gaussian blur—applying isotropic smoothing, with kernel size and $\left(\sigma_{x},\sigma_{y}\right)$ determined by the sampled magnitude. These perturbations mimic subtle variations in scanner sharpness or acquisition noise without distorting hemorrhage morphology.

Using the described strategies, validation metrics were evaluated at the three predefined augmentation levels:

- Level 0: No augmentation;
- Level 1: Affine transformations only (TrivialAugment-based spatial augmentations);
- Level 2: Affine transformations + pixel-value transformations (Gaussian noise and Gaussian blur, applied probabilistically).

### 3.4. Model Architecture

**Figure 3:**
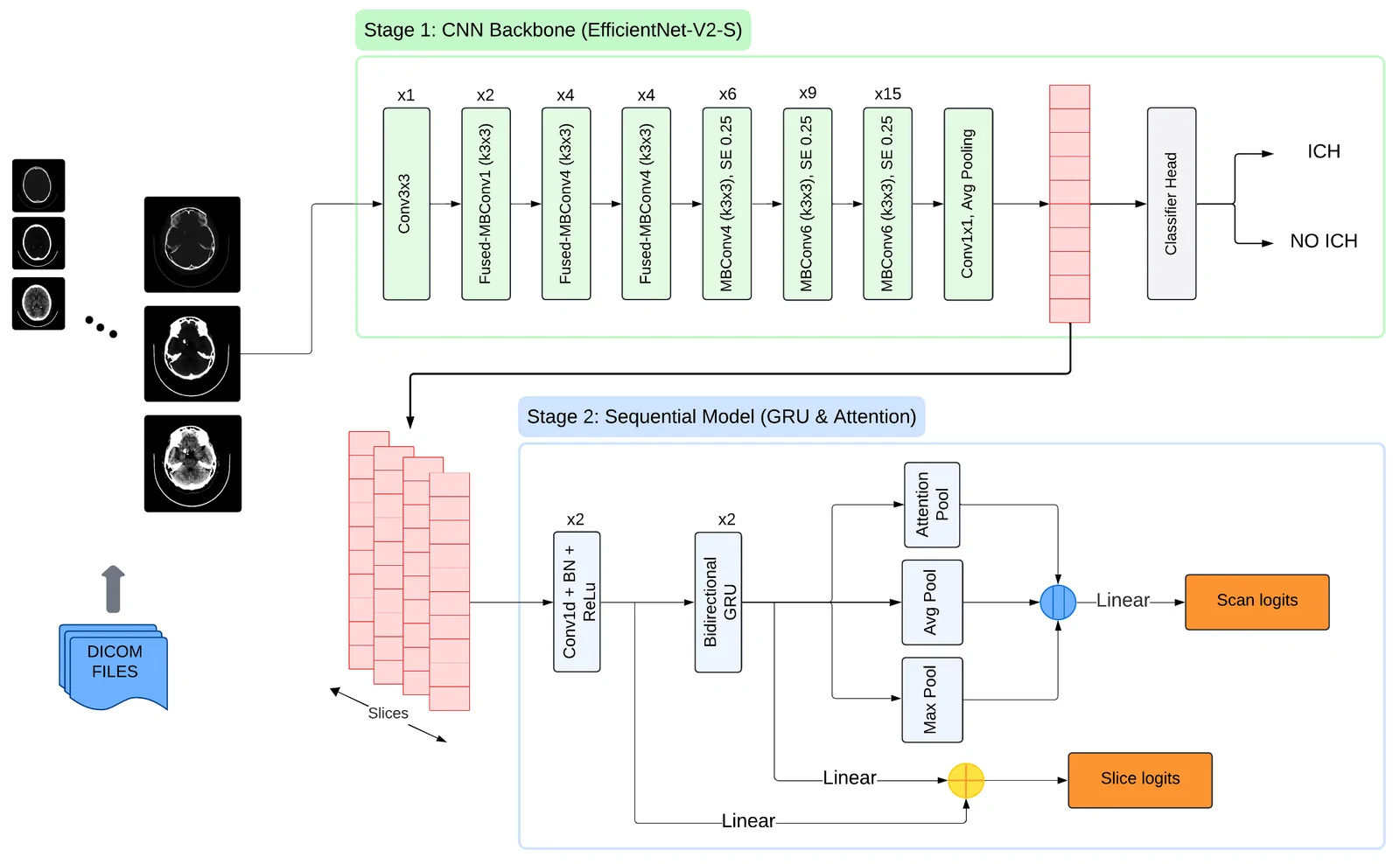
Overall architecture for the hybrid ICH detection framework from brain CT scans. Individual DICOM slices are first processed through a convolutional backbone (EfficientNet-V2-S), which extracts per-slice feature embeddings. These embeddings are then passed to a sequential module composed of convolutional layers, bidirectional GRUs, and multi-pooling (attention, average, and max). The model produces both slice-level logits and an aggregated scan-level prediction.

As observed in Figure 3, the proposed framework consists of two sequential stages: CNN backbone training and sequence model training. In the first stage, the CNN backbone serves as a slice-level feature extractor, generating rich representations and predicting the probability of ICH for each isolated slice through a classifier head. In the second stage, the sequence module is introduced for optimizing scan-level study aggregation while concurrently refining slice-level characterizations. By explicitly processing the full sequence of slice features in each scan, this stage enables the framework to transition from isolated slice evaluations to a global architecture that leverages anatomical and contextual inter-slice dependencies.

#### 3.4.1. Backbone Architecture

The selection of an appropriate CNN backbone is critical for balancing feature extraction quality with computational efficiency. To identify a suitable architecture, we compared pretrained convolutional models available in PyTorch TorchVision using the official ImageNet-1K top-1 and top-5 accuracy metrics and parameter counts reported by the library [54,55]. The architectures included in this comparison correspond to models adopted in the literature reviewed and others with a parameter scale comparable to the selected backbone, enabling a meaningful assessment of performance–efficiency trade-offs. The comparison is summarized in Table 7.

EfficientNet-V2-S was selected as the CNN backbone for this study due to its favorable balance between classification performance and model size. As shown in Table 7, it achieves 84.2% top-1 accuracy on ImageNet-1K with only 21.5 million parameters—substantially fewer than larger architectures such as ViT-L/16 (304.3 M parameters) or ResNeXt-101 (88.8 M parameters) while maintaining competitive accuracy. Among models with similar parameter counts, EfficientNet-V2-S outperforms alternatives such as ResNet-34 (73.3% accuracy, 21.8 M parameters) and demonstrates superior efficiency compared to DenseNet variants with lower accuracy despite having fewer parameters.

The clinical relevance of the EfficientNet family in this specific domain is further underscored by the recent literature. For instance, Malik et al. [56] evaluated various architectures for ICH diagnosis, demonstrating that EfficientNet-B3—a representative of the older, first-generation (V1) family with $\tilde{1}2.2$ M parameters—achieved a superior validation accuracy of 93.29%, outperforming larger and structurally diverse models such as ResNet-50 and ResNeXt-50. This highlights the strong domain-specific feature extraction capabilities inherited by the family, though the legacy V1 design is known to introduce training bottlenecks on modern hardware due to intensive early depthwise convolutions [57].

To address these limitations, the EfficientNetV2 architecture introduces optimizations designed for faster training and improved parameter efficiency compared to previous CNN generations [57]. This aligns with the objectives of the present study by providing a state-of-the-art architecture with rapid training and inference times, which are critical in medical workflows requiring timely model updates and fast predictions.

Specifically, EfficientNetV2-S incorporates several architectural innovations to achieve these efficiency gains. Early layers predominantly use Fused-MBConv (Mobile Inverted Bottleneck Convolution) blocks, which merge the operations of standard MBConv blocks [58] into a single convolution, improving accelerator utilization. Smaller expansion ratios and 3 × 3 kernel sizes throughout the network reduce memory and computational overhead, while additional layers preserve sufficient receptive fields. The architecture also integrates Squeeze-and-Excitation (SE) modules to enhance feature representation. With approximately 22 million parameters and 8.8 billion FLOPs, these combined mechanisms allow EfficientNetV2-S to maximize representation capability while demonstrating significantly faster training throughput than comparable baseline models.

A transfer learning strategy was employed, fine-tuning all pretrained model weights. The EfficientNetV2-S weights were obtained from PyTorch TorchVision (version 0.23.0; PyTorch Foundation, San Francisco, CA, USA) [55], where the model was originally trained on the ImageNet-1K dataset.

All experiments were conducted for 42 epochs using the Adam optimizer [59] with a learning rate of $5\times 10^{-4}$, a batch size of 32, and a weight decay of $5\times 10^{-5}$. A cosine annealing learning rate scheduler was used to gradually decrease the learning rate following a cosine decay pattern. A learning rate warm-up of 5 epochs and dropout regularization in the classifier head were also evaluated.

The model was trained using the cross-entropy loss function to optimize the prediction of ICH presence at the slice level, formulated as a two-class classification problem. Model weights were updated based on the training set, while the best model checkpoint was selected according to the lowest validation loss, monitored at each epoch.

To accelerate training, Automatic Mixed Precision (AMP) [60] with gradient scaling was employed, improving computational efficiency while preserving numerical stability and model accuracy.

#### 3.4.2. Sequence Modeling

In the second stage, a sequence model is trained on the features extracted by the backbone, explicitly processing the sequence of slice features for each scan in a manner that mirrors how clinicians interpret complete CT volumes. A learned positional encoding is added to the slice features prior to sequential modeling, allowing the network to retain information about each slice’s spatial location within the scan. This architecture is designed to maximize the accuracy and calibration of scan-level ICH detection by capturing global volumetric context, while simultaneously enforcing structural constraints that smooth and refine underlying slice-level predictions.

To model these inter-slice dependencies, a bidirectional Gated Recurrent Unit (GRU) with two layers and a hidden dimension of 96 is employed, allowing contextual information from both preceding and succeeding slices. This is further refined by an attention-based mechanism that adaptively weights the slice representations, emphasizing the contribution of relevant regions for scan-level detection.

An additional data augmentation strategy is applied at the sequence level: during training, only a subset of slices is provided to the sequence model using a random subsampling approach inspired by [24]. While their method samples 25 slices without preserving their spatial arrangement, we maintain temporal order when subsampling. Through hyperparameter tuning, we selected 30 slices as the optimal subset size. This strategy improves robustness to variable scan lengths and helps the model generalize to incomplete or heterogeneous studies.

As shown in Figure 3, the model follows a dual-branch design to execute both scan-level and slice-level tasks. For scan-level prediction, the model relies on the GRU-derived sequence representations. These features are aggregated through three complementary pooling mechanisms designed to capture diverse macro-level information: max pooling to isolate the most salient activations, average pooling to encode global scan trends, and attention-weighted pooling to adaptively emphasize diagnostically critical slices. The pooled outputs are concatenated and passed through a final classifier to produce the primary scan-level ICH prediction.

Concurrently, for the secondary slice-level branch, the architecture maintains a dual-path classification setup to capture localized dependencies. The first path preserves direct slice-wise classification using the reduced backbone features, while the second path routes these features through the bidirectional GRU to inject sequential context. The final slice-level output is obtained by combining the logits from both paths via a residual connection, enabling the framework to optimize localized predictions using both immediate slice characteristics and neighboring contextual information.

The model is trained using a dual-level loss function that jointly optimizes slice-level and scan-level predictions:

(1) $$L_{\mathrm{total}}=\lambda_{\mathrm{slice}}\;L_{\mathrm{slice}}+\lambda_{\mathrm{scan}}\;L_{\mathrm{scan}}$$

where $\lambda_{\mathrm{slice}}$ and $\lambda_{\mathrm{scan}}$ control the relative importance of each objective. We employ the standard binary cross-entropy (BCE) loss at both levels:

(2) $$L_{\mathrm{BCE}}=-\frac{1}{N}\sum_{i=1}^{N}\left[y_{i}\log\left(\sigma (z_{i})\right)+\left(1-y_{i}\right)\log\left(1-\sigma (z_{i})\right)\right]$$

where $y_{i}$ is the true label, $z_{i}$ is the model’s logit output, and σ denotes the sigmoid activation function.

To determine the optimal weighting scheme, we evaluated three configurations: balanced weighting ($\lambda_{\mathrm{slice}}=\lambda_{\mathrm{scan}}=1.0$), slice-focused weighting ($\lambda_{\mathrm{slice}}=2.0,\lambda_{\mathrm{scan}}=1.0$), and scan-focused weighting ($\lambda_{\mathrm{slice}}=1.0,\lambda_{\mathrm{scan}}=2.0$). As detailed in Section 4, the balanced configuration was selected based on performance across both levels.

To optimize the sequence model, we use the Adam optimizer with an initial learning rate of $4\times 10^{-4}$. Training employs a Reduce-on-Plateau scheduler (patience = 5, factor = 0.7) to automatically lower the learning rate when validation performance stabilizes. Training is performed for 50 epochs using a batch size of 128.

#### 3.4.3. Resource Utilization

The convolutional backbone was trained on Amazon Web Services (Amazon Web Services, Inc., Seattle, WA, USA) using a g4dn.xlarge instance, with an NVIDIA T4 GPU (NVIDIA Corporation, Santa Clara, CA, USA) with 16 GB of VRAM, 4 vCPUs based on the Intel Xeon Scalable (Cascade Lake) family (Intel Corporation, Santa Clara, CA, USA), and 16 GB of system memory.

For the sequence model, training was conducted on a workstation equipped with an NVIDIA RTX 4060 GPU (NVIDIA Corporation, Santa Clara, CA, USA) (8 GB VRAM), 32 GB of RAM, and a 13th Gen Intel(R) Core(TM) i9-13900H CPU (Intel Corporation, Santa Clara, CA, USA).

### 3.5. Evaluation Protocol

The performance of the proposed model was assessed through complementary internal and external validation strategies to evaluate both within-distribution performance and cross-domain generalization.

#### 3.5.1. Internal Validation on RSNA Test Set

Internal validation was conducted on the independent RSNA test set described in Table 3, comprising 1000 studies (34,483 slices) held out during training and validation. This evaluation assesses performance under the same data distribution used for model development and provides insight into generalization within the RSNA multi-institutional setting.

Both scan-level and slice-level performance were evaluated.

#### 3.5.2. External Validation on PhysioNet Dataset

External validation was performed on the PhysioNet ICH dataset to evaluate model robustness across different institutions, patient populations, acquisition protocols, and geographic regions. The complete dataset of 75 publicly available CT studies was used, with no overlap between training and evaluation data.

Performance was evaluated at both the study and slice levels. Study-level metrics represent the primary clinically relevant endpoint for automated ICH triage, while slice-level evaluation provides additional insight into the transferability of learned localization-related representations across imaging protocols.

#### 3.5.3. External Validation on CQ500 Dataset

External validation was additionally performed on the CQ500 dataset to evaluate generalization under substantial acquisition differences, particularly the predominance of thin-slice reconstructions relative to the RSNA training data.

Scan-level evaluation was conducted on all 472 studies using the majority-vote reference labels described in Section 3.1.3. Since the original CQ500 release does not provide slice-level annotations, slice-level evaluation was restricted to a label-consistent subset of 416 studies for which BHX-derived slice annotations were available and produced a scan-level signal consistent with the CQ500 majority-vote labels. Slice-level metrics on CQ500 are additionally reported to provide complementary insight into model behavior at finer anatomical granularity.

#### 3.5.4. Performance Metrics

Model performance was quantified using multiple complementary metrics: Area Under the Receiver Operating Characteristic Curve (AUROC), Area Under the Precision-Recall Curve (AUPRC), Accuracy, Precision, Recall (Sensitivity), and F1-score.

AUROC measures the probability that a randomly selected positive instance receives a higher prediction score than a randomly selected negative instance and provides threshold-independent assessment of discrimination ability.

AUPRC summarizes the trade-off between Precision and Recall across decision thresholds and is particularly informative under class imbalance. AUPRC focuses specifically on the model’s performance on the positive class, making it a more informative metric when the positive class is rare or when false positives and false negatives carry different clinical costs.

Accuracy measures the proportion of correctly classified instances:

(3) $$\mathrm{Accuracy}=\frac{TP+TN}{TP+TN+FP+FN}$$

Precision (Positive Predictive Value) measures the proportion of positive predictions that are correct:

(4) $$\mathrm{Precision}=\frac{TP}{TP+FP}$$

Recall (Sensitivity) measures the proportion of positive instances correctly identified:

(5) $$\mathrm{Recall}=\frac{TP}{TP+FN}$$

The F1-score provides the harmonic mean between Precision and Recall:

(6) $$F1=2\times \frac{\mathrm{Precision}\times \mathrm{Recall}}{\mathrm{Precision}+\mathrm{Recall}}$$

where TP, TN, FP, and FN denote true positives, true negatives, false positives, and false negatives, respectively.

For threshold-dependent metrics (Accuracy, Precision, Recall, and F1-score), predictions were binarized using a decision threshold of 0.5. Confusion matrices were additionally reported to provide a direct visualization of classification outcomes and error distributions.

To compare the discrimination performance of the backbone and full hybrid model, AUROC values were compared using DeLong’s method [61], a nonparametric test for assessing differences between correlated ROC curves. For scan-level evaluation, the backbone model generated slice-level predictions, and the scan-level probability was defined as the maximum predicted probability across all slices within the study. Scan-level AUROCs were then compared using the standard nonparametric DeLong test. For slice-level evaluation, comparisons were reported using both the standard DeLong formulation and a cluster-aware extension that accounts for within-scan correlation among slices originating from the same study. The implementation follows the computationally efficient exact formulation proposed by Sun et al. [62]. Statistical significance was assessed at α=0.05.

## 4. Results

This section reports the performance of the proposed hybrid ICH detection framework across slice-level and scan-level tasks on three datasets of increasing distributional distance from the training data: the held-out RSNA test set, the external PhysioNet cohort, and the external CQ500 cohort. CNN backbone windowing and augmentation strategies are first compared to support the backbone training decisions described in Section 3. Dual-level loss weighting decisions are then compared to support the sequence module training configuration. Having established the final model configurations, model performance is evaluated on both internal and external validation datasets. Internal validation is first performed on RSNA, followed by external validation on PhysioNet and CQ500. For each dataset, results are reported at both the scan and slice levels, combining threshold-independent discrimination, evaluated through AUROC, AUPRC, and DeLong’s test, with threshold-dependent metrics and full confusion matrices. These complementary perspectives address different clinical questions: AUROC and AUPRC characterize ranking quality for prioritization workflows in which every scan is ultimately reviewed by a specialist but reordered according to predicted probability, whereas threshold-dependent metrics characterize behavior under the fixed-threshold alerting paradigm used by most current triage systems.

### 4.1. CNN Backbone: Windowing and Augmentation Strategy on RSNA

Prior to integrating the sequence module, several preprocessing and augmentation configurations for the CNN backbone were evaluated using the RSNA dataset.

The windowing strategy and augmentations were found to be important factors affecting model performance. Although additional hyperparameters were explored during training, none produced meaningful improvements beyond the final setup. Table 8 compares the final configuration—sigmoid multi-windowing with Level 2 augmentation (affine and pixel-value transformations)—against a baseline model using a single-window strategy without augmentation. While the baseline model demonstrates the ability to learn ICH-related patterns, achieving an AUROC of approximately 0.80, its overall performance is substantially lower across all metrics. These results highlight the importance of both the preprocessing and augmentation strategies adopted in this study.

**Figure 4:**
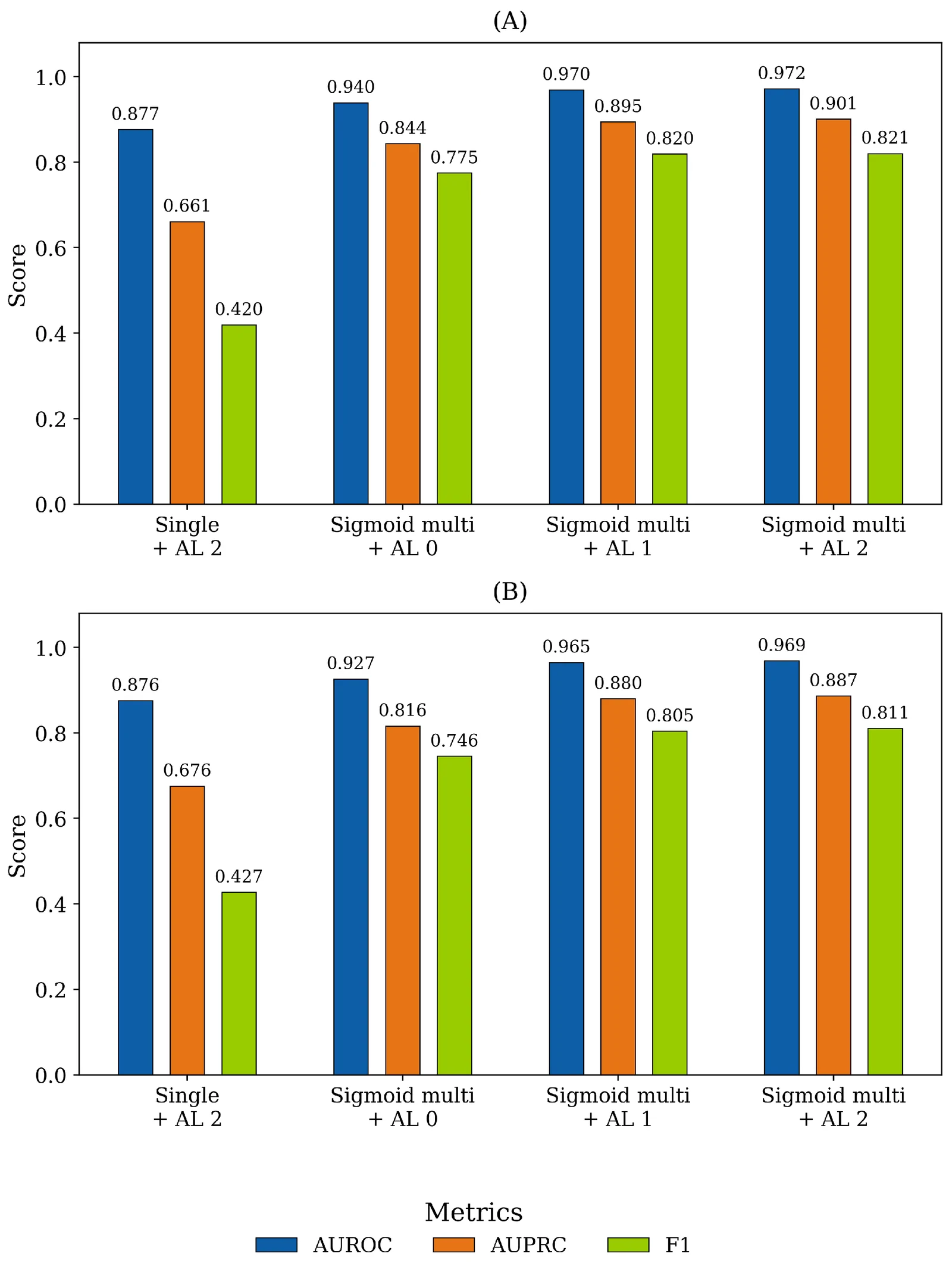
Slice-level performance metrics (AUROC, AUPRC, and F1-Score) of the CNN backbone under different windowing and augmentation configurations. (**A**) Test set metrics. (**B**) Validation set metrics. From left to right, the first configuration corresponds to the single-window strategy evaluated with Level 2 augmentation, followed by the sigmoid multi-window strategy evaluated with augmentation levels 0, 1, and 2, respectively.

To better understand the relative contribution of each component, Figure 4 compares performance across multiple windowing and augmentation configurations. Windowing decisions had the strongest impact on model performance. As observed in Figure 4, the performance of a model using the sigmoid multi-window strategy does not degrade substantially when no augmentation is applied, compared to the full Level 2 augmentation setup. Relative to the no-augmentation setting, the addition of Level 2 augmentation improves AUROC, AUPRC, and F1-score on the test set by 3.5%, 6.8%, and 5.8%, respectively. In contrast, the performance gains obtained by adding pixel-value augmentations on top of affine transformations are comparatively modest, suggesting that affine transformations capture most of the augmentation benefit.

Notably, Figure 4 also shows that even when trained with the strongest augmentation configuration, the single-window model underperforms the sigmoid multi-window strategy without any augmentation across all metrics. This observation suggests that, in this setting, the windowing strategy may contribute more substantially to performance improvements than augmentation, though both appear to play a meaningful role in the CNN backbone’s overall performance.

Based on these findings, the sigmoid multi-window strategy combined with Level 2 augmentation was selected as the final backbone configuration for subsequent experiments. As shown in Table 8, the resulting validation and test metrics remain closely aligned, supporting the model selection procedure based on validation loss and indicating good generalization to held-out RSNA data.

### 4.2. Hybrid Model: Dual-Level Loss Weighting on RSNA

Following the selection of the final CNN backbone configuration, the next step involved determining how the slice-level and scan-level objectives should be weighted when training the complete hybrid model. Because the sequence module is trained with a dual-level loss that combines a slice-level term and a scan-level term, the relative weighting of these two objectives, $\lambda_{\mathrm{slice}}$ and $\lambda_{\mathrm{scan}}$, can be expected to affect which level the model prioritizes during optimization.

Three weighting configurations were evaluated: balanced weights ($\lambda_{\mathrm{slice}}=\lambda_{\mathrm{scan}}=1.0$), scan-focused weights ($\lambda_{\mathrm{slice}}=1.0$, $\lambda_{\mathrm{scan}}=2.0$), and slice-focused weights ($\lambda_{\mathrm{slice}}=2.0$, $\lambda_{\mathrm{scan}}=1.0$). Table 9 compares scan-level and slice-level performance across all three configurations on the RSNA test set. Across both evaluation levels, the balanced configuration achieved the lowest BCE loss (0.181 scan-level; 0.126 slice-level), whereas both focused configurations produced higher loss values. Although the scan-focused configuration achieved marginally higher scan-level AUROC and AUPRC, these gains were small and accompanied by lower scan-level precision and F1-score, together with slight reductions in slice-level AUPRC and F1-score. Conversely, the slice-focused configuration produced the largest decrease in scan-level F1-score.

The balanced configuration provided the best overall trade-off between scan-level and slice-level performance, avoiding the scan-level F1-score reductions observed with the focused configurations, while also avoiding the slice-level degradation seen under scan-focused weighting. Although scan-level prediction remains the primary clinically actionable output of the proposed framework, slice-level supervision provides dense supervisory signals that support stable optimization and preserve localized representations for future localization-oriented extensions. Considering both optimization loss and predictive performance at the scan and slice levels, the balanced weighting configuration ($\lambda_{\mathrm{slice}}=\lambda_{\mathrm{scan}}=1.0$) was adopted as the final hybrid model configuration used throughout the remainder of this work. Table 10 reports its scan-level performance on the RSNA validation and test sets. As with the CNN backbone, validation and test metrics remain closely aligned (AUROC: 0.980 test vs. 0.983 validation; F1-score: 0.927 on both partitions), confirming that the dual-level objective generalizes well to held-out data and that the scan-level comparisons reported in the remainder of this section are not confounded by overfitting.

### 4.3. Internal Validation (RSNA)

Having established the final backbone and hybrid model configurations, both models were evaluated on the held-out RSNA test set using the protocol described in Section 3.5. Results are reported at both the scan and slice levels, combining threshold-independent discrimination metrics (AUROC, AUPRC, and DeLong’s test) with threshold-dependent performance measures and confusion matrices. For the CNN backbone, scan-level probability was defined as the maximum predicted probability across all slices within a study, whereas the hybrid model directly produces both scan-level and slice-level predictions through its dual-level architecture. This evaluation provides a complete characterization of model behavior on data taken from the same distribution used during model development before proceeding to external validation.

At the scan level, the hybrid model achieved an AUROC of 0.980 and an AUPRC of 0.977, compared to 0.977 and 0.975 for the backbone (Table 11). This improvement was found to be statistically significant (p=0.039) via the DeLong test. At the predefined classification threshold, the hybrid model demonstrated a shift toward high-precision behavior. The confusion matrix reveals a sharp reduction in false positives from 71 to 23, driving precision up from 0.846 to 0.942. While this gain was accompanied by a minor increase in false negatives (from 19 to 36) that lowered recall from 0.953 to 0.912, the net effect on overall classification performance was positive. Overall accuracy improved from 0.910 to 0.941, and F1-score increased from 0.896 to 0.927. These results indicate that the sequence module refines ranking quality while improving overall classification performance.

At the slice level, the hybrid model achieved an AUROC of 0.981 and an AUPRC of 0.923, compared to 0.972 and 0.901 for the backbone (Table 12). Under a standard DeLong test treating all slices as independent, this difference was significant ($p=2.90\times 10^{-63}$). When accounting for within-scan correlation using a clustered DeLong test (n=1000 clusters), the AUROC difference did not reach statistical significance (p=0.241). At the operational threshold, the hybrid model demonstrated diagnostic improvements. The model simultaneously reduced both false positives (from 667 to 573) and false negatives (from 1051 to 987). This dual optimization translated into uniform metric gains: precision increased from 0.855 to 0.875, recall from 0.789 to 0.802, and F1-score rose from 0.821 to 0.837.

**Figure 5:**
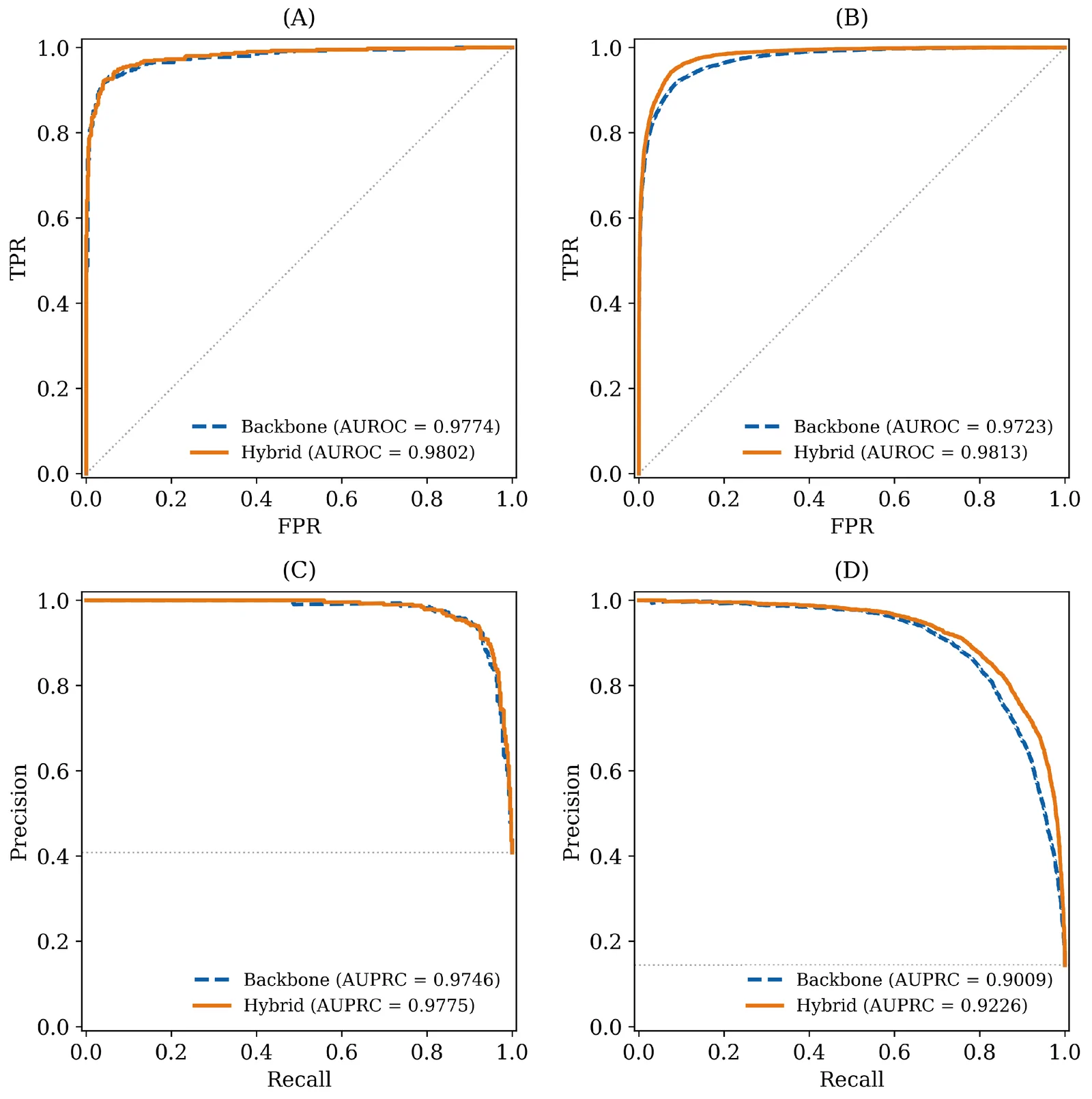
ROC and PR curves comparing the CNN backbone and hybrid model on the RSNA test set. (**A**) Scan-level ROC curves. (**B**) Slice-level ROC curves. (**C**) Scan-level PR curves. (**D**) Slice-level PR curves. The dotted diagonal in (**A**,**B**) denotes chance-level performance; the dotted horizontal line in (**C**,**D**) denotes the positive-class prevalence.

Corresponding ROC and precision–recall (PR) curves for the CNN backbone and hybrid model on the RSNA test set are shown in Figure 5.

### 4.4. External Validation

External validation was conducted on the PhysioNet and CQ500 datasets using the final backbone and hybrid model configurations, without retraining. Both datasets differ from RSNA in acquisition protocol, patient population, and geographic origin, and CQ500 additionally introduces a substantial shift in slice thickness relative to the training data, as described in Section 3.1.3.

#### 4.4.1. PhysioNet

At the scan level, the hybrid model achieved an AUROC of 0.914 and an AUPRC of 0.932, compared to 0.900 and 0.921 for the backbone (Table 13). This difference did not reach statistical significance (p=0.186) via the DeLong test. At the predefined classification threshold, however, the hybrid model showed a shift toward higher-precision behavior. The confusion matrix reveals a reduction in false positives from 25 to 6, raising precision from 0.583 to 0.829. This gain was accompanied by a minor increase in false negatives (from 1 to 7) that lowered recall from 0.972 to 0.806. Overall accuracy increased from 0.653 to 0.827, and F1-score increased from 0.729 to 0.817.

At the slice level, the hybrid model achieved an AUROC of 0.960 and an AUPRC of 0.836, compared to 0.951 and 0.813 for the backbone (Table 14). Under a standard DeLong test treating all slices as independent, this difference was significant ($p=7.71\times 10^{-5}$). When accounting for within-scan correlation using a clustered DeLong test (n=75 clusters), the AUROC difference did not reach statistical significance (p=0.781). At the operational threshold, the hybrid model showed a consistent numerical improvement across precision, recall, and F1-score. The model simultaneously reduced both false positives (from 154 to 88) and false negatives (from 76 to 72). This translated into uniform numerical gains: precision increased from 0.611 to 0.737, recall increased from 0.761 to 0.774, and F1-score rose from 0.678 to 0.755, consistent with the trend observed at the scan level.

**Figure 6:**
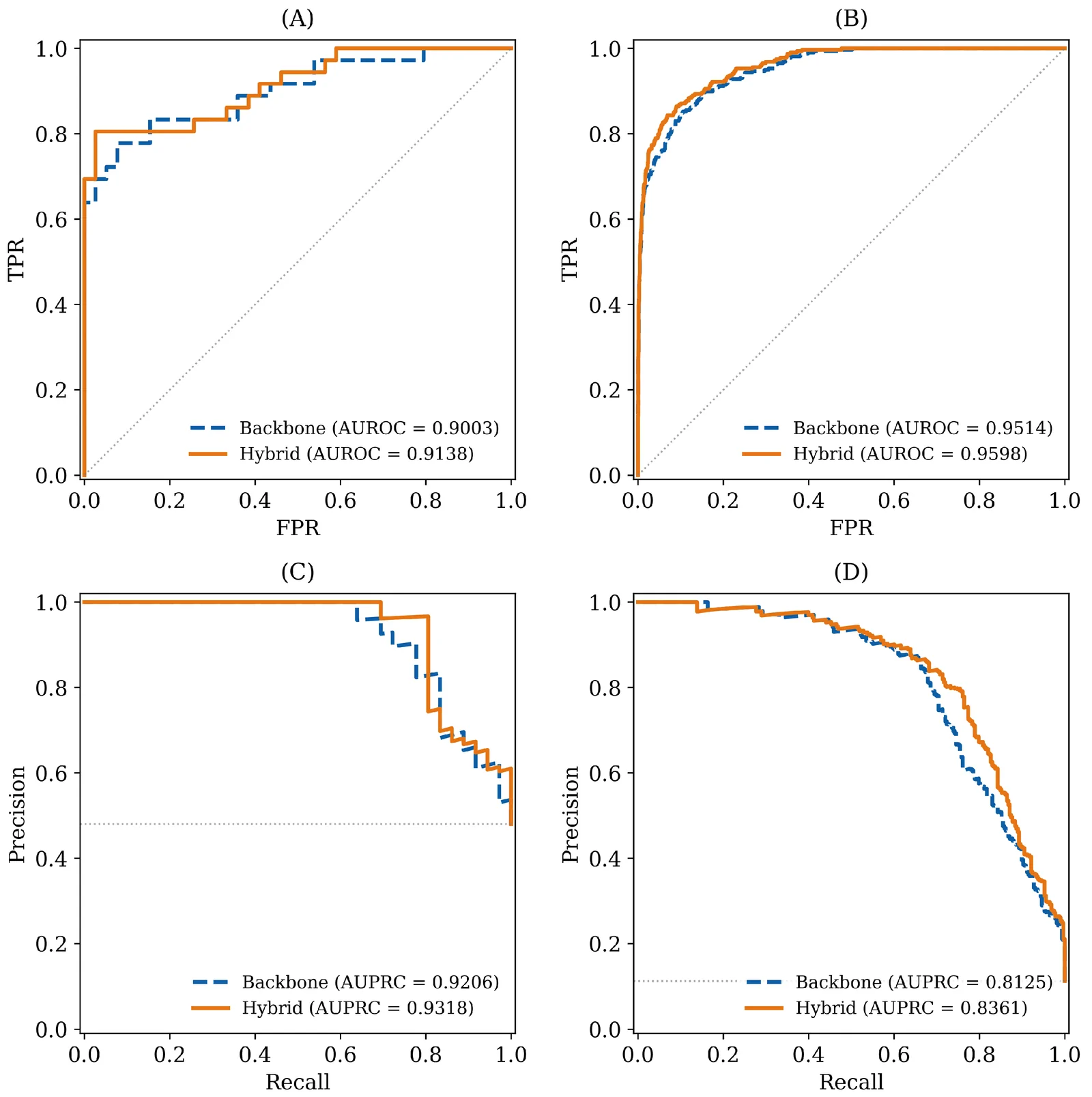
ROC and PR curves comparing the CNN backbone and hybrid model on the external PhysioNet cohort. (**A**) Scan-level ROC curves. (**B**) Slice-level ROC curves. (**C**) Scan-level PR curves. (**D**) Slice-level PR curves. The dotted diagonal in (**A**,**B**) denotes chance-level performance; the dotted horizontal line in (**C**,**D**) denotes the positive-class prevalence.

Corresponding ROC and PR curves for the CNN backbone and hybrid model on the PhysioNet cohort are shown in Figure 6.

#### 4.4.2. CQ500

External validation on CQ500 was performed using the 472 retained studies described in Section 3.1.3 for scan-level evaluation, and the 416-study agreement subset for slice-level evaluation, for which BHX-derived slice labels are consistent with the majority-vote scan-level reference.

Evaluating performance at the scan level, the hybrid model yielded an AUROC of 0.905 and an AUPRC of 0.909, showing a marginal gain over the backbone’s metrics of 0.900 and 0.906 (Table 15). This increment in ranking performance, however, lacked statistical significance according to the DeLong test (p=0.339). At the decision threshold, classification behavior changed. The hybrid architecture reduced the backbone’s false positives from 167 to 57 and increased precision from 0.526 to 0.748. This shift led to 16 additional false negatives (moving from 12 to 28) and a corresponding drop in recall from 0.939 to 0.858. At this fixed operating point, overall accuracy increased from 0.621 to 0.820, and F1-score increased from 0.674 to 0.799.

At the slice level, the hybrid model achieved an AUROC of 0.937 and an AUPRC of 0.772, outpacing the backbone estimates of 0.928 and 0.758 (Table 16). Under a standard DeLong test treating all slices as independent, this difference was significant ($p=4.14\times 10^{-97}$). When accounting for within-scan correlation using a clustered DeLong test (n=416 clusters), the AUROC difference did not reach statistical significance (p=0.921). At the operational threshold, the hybrid model showed a numerical improvement, consistent with the pattern observed on the other datasets in this study. The model reduced false positives from 1843 to 1518, though it experienced a minor increase in false negatives (from 4021 to 4073). This trade-off corresponded to precision increasing from 0.797 to 0.826 and overall accuracy rising slightly from 0.935 to 0.938, with a corresponding F1-score increase from 0.712 to 0.720.

**Figure 7:**
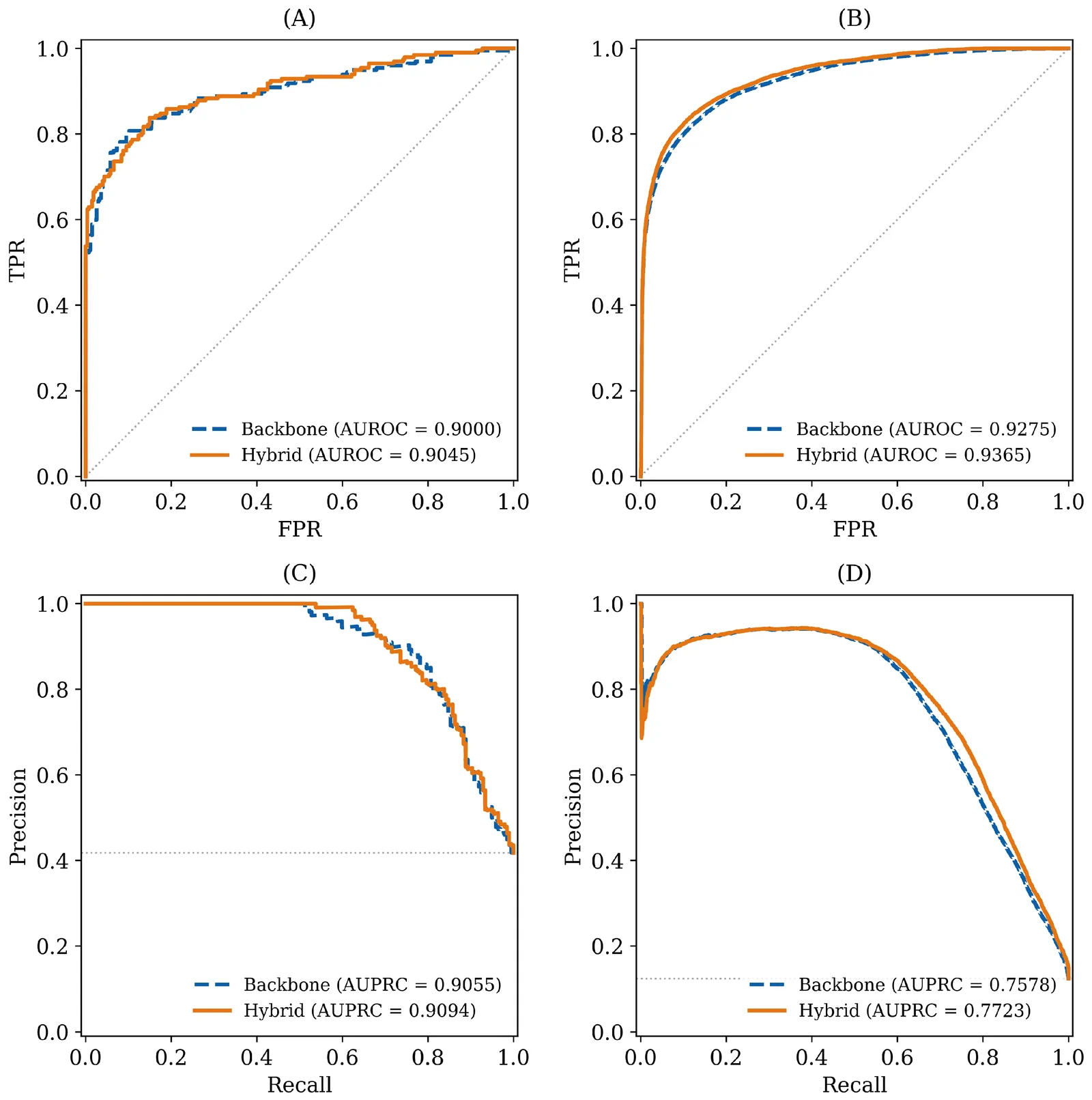
ROC and PR curves comparing the CNN backbone and hybrid model on the external CQ500 cohort. (**A**) Scan-level ROC curves. (**B**) Slice-level ROC curves. (**C**) Scan-level PR curves. (**D**) Slice-level PR curves. The dotted diagonal in (**A**,**B**) denotes chance-level performance; the dotted horizontal line in (**C**,**D**) denotes the positive-class prevalence.

Corresponding ROC and PR curves for the CNN backbone and hybrid model on the CQ500 cohort are shown in Figure 7.

### 4.5. Summary of Cross-Dataset Performance

The cumulative evaluation across the three cohorts demonstrates the ability of the proposed hybrid architecture to generalize beyond the training distribution. When transitioning from the internal RSNA test set to the external PhysioNet and CQ500 datasets, an expected reduction in performance was observed due to distributional differences. Nevertheless, the hybrid model maintained strong discrimination performance, achieving scan-level AUROCs of 0.980 on RSNA, 0.914 on PhysioNet, and 0.905 on CQ500. Across all three cohorts, the hybrid architecture consistently matched or exceeded the backbone’s discrimination performance.

While the sequence module was primarily introduced to model inter-slice dependencies and improve scan-level decision making, its benefits extended to both evaluation levels. Across all three cohorts, the hybrid model improved scan-level classification performance, particularly by reducing false-positive study assignments and increasing precision. Improvements in ranking performance were also observed across all datasets, although the statistical significance of the AUROC gain varied by cohort size, reaching significance on the large internal RSNA test set (p=0.039) but not on the external PhysioNet (p=0.186) and CQ500 (p=0.339) cohorts. Nevertheless, at the operational classification threshold, the hybrid model consistently showed improved scan-level classification behavior across all three datasets. These improvements were driven by marked reductions in false-positive scan assignments, which increased scan-level precision by +0.096 on RSNA, +0.245 on PhysioNet, and +0.222 on CQ500, leading to corresponding improvements in both overall accuracy and F1-score.

At the slice level, although clustered DeLong testing did not identify statistically significant AUROC differences between the backbone and hybrid models on any dataset, the hybrid architecture consistently produced higher AUPRC, precision, and F1-scores than the backbone. In addition, the model reduced both false-positive and false-negative slice assignments on the RSNA and PhysioNet cohorts, while substantially reducing false positives on CQ500 with only a minor increase in false negatives. Collectively, these results indicate that the incorporation of inter-slice contextual information provides operational benefits across both internal and external validation datasets.

## 5. Discussion

### 5.1. Model Performance and Architectural Contributions

The proposed resource-efficient hybrid model achieved strong performance on the RSNA held-out test set, with scan-level AUROC of 0.980 and AUPRC of 0.977. These results demonstrate that the two-stage architecture—comprising an efficient CNN backbone for slice-level feature extraction followed by a bidirectional GRU for sequential modeling—effectively captures both local hemorrhage patterns and inter-slice anatomical dependencies.

The preprocessing pipeline and augmentation strategy proved effective, as evidenced by the close alignment between validation and test metrics at both slice and scan levels. As shown in Section 4.1 (Table 8, Figure 4), the sigmoid multi-window strategy appeared to be one of the strongest contributors to performance improvements: even without augmentation, models trained with sigmoid multi-windowing substantially outperformed single-window approaches with strong augmentation. These results suggest that, in this setting, the windowing strategy may contribute more substantially to performance improvements than augmentation, though both appear to play a meaningful role in the CNN backbone’s overall performance.

The dual-level training objective enabled simultaneous optimization of slice-level and scan-level predictions. As shown in Section 4.2, balanced loss weighting ($\lambda_{\mathrm{slice}}=\lambda_{\mathrm{scan}}=1.0$) achieved the lowest optimization loss at both evaluation levels and avoided the precision and F1-score trade-offs observed under the focused weighting schemes. This finding aligns with observations in the literature [16], suggesting that equal weighting offers an effective compromise between clinically relevant scan-level performance and the preservation of informative slice-level representations.

The addition of the sequence module produced consistent improvements over the CNN backbone across all three datasets and both evaluation levels. While the scan-level AUROC gain reached statistical significance only on the internal RSNA cohort (p=0.039), the external cohorts showed the same directional improvement in AUROC and AUPRC, although these differences were not statistically significant. The most consistent gains were observed in threshold-dependent metrics, with reductions in false-positive scan classifications leading to improved precision, accuracy, and F1-score across all datasets. These improvements were particularly pronounced on the external cohorts, where scan-level precision increased by +0.245 on PhysioNet and +0.222 on CQ500. Collectively, these findings suggest that inter-slice contextual modeling primarily refines the model’s operating characteristics at a fixed decision threshold, providing practical benefits even when improvements in ranking performance are modest.

### 5.2. Generalization Across Heterogeneous Clinical Datasets

External validation was conducted on two independent datasets representing distinct forms of distribution shift from the RSNA training data, substantially strengthening the generalizability evidence relative to the original manuscript.

The two external cohorts represent complementary forms of distribution shift. PhysioNet introduces geographic, institutional, and demographic variation, reflecting a single-center emergency setting in Iraq with a predominantly younger trauma population, in contrast to RSNA’s multi-institutional composition. CQ500 additionally introduces a technical distribution shift through its predominance of thin-slice (0.625 mm) reconstructions relative to the 3–5 mm slice thickness used in training. Despite these differences, the hybrid model maintained strong discrimination performance across both external datasets and preserved the same pattern of threshold-dependent improvement observed internally.

Wang et al. [24] evaluated their approach on the PhysioNet dataset, achieving a slice-level AUROC of 0.964 using an ensemble of three large backbones (approximately 110 M parameters total) trained on 19,530 studies. The present framework achieved a comparable AUROC of 0.960 using a single 22M-parameter backbone trained on 6000 studies, though Wang et al. reported higher sensitivity at the default threshold (0.887 vs. 0.774), a gap attributable to their larger training set and ensemble design. A full comparison across threshold-dependent metrics is not possible, as Wang et al. reported only AUROC, sensitivity, and specificity.

### 5.3. Practical Applicability, Reproducibility, and Resource Constraints

Beyond detection performance, this work addresses practical constraints that limit the reproducibility and independent development of DL systems for ICH detection. Computational efficiency is treated not merely as a deployment consideration, but as a prerequisite for sustainable model training, exploration, and institutional ownership, particularly in resource-constrained clinical and research environments.

The proposed framework achieves competitive performance with substantially reduced computational requirements. Using a compact EfficientNetV2-S backbone and training on only 6000 stratified studies (28% of the RSNA dataset), the complete framework can be trained in under 26 h on a single GPU while maintaining performance competitive with substantially larger architectures. This substantially lowers the computational barrier for independent retraining, validation, and adaptation by research institutions, particularly in resource-constrained settings.

From a clinical perspective, the scan-level prediction design aligns with radiological workflows, where decisions are made at the study level. In high-volume emergency settings and environments with limited specialist coverage, automated scan-level ICH detection can support case prioritization and preliminary assessment, while preserving the radiologist’s role as the final decision-maker. Together, these characteristics support practical deployment in diverse healthcare settings and reduce structural dependency on externally trained, computationally intensive models.

### 5.4. Comparison with Existing ICH Detection Literature

Table 17 contextualizes the proposed method relative to representative deep learning approaches for ICH detection. The proposed method’s contribution is not reducible to any single architectural choice; rather, it reflects a combination of properties that are rarely present simultaneously in existing work: computational efficiency, explicit scan-level supervision, inter-slice contextual modeling, dual-level evaluation, and multi-cohort external validation.

Existing approaches in the ICH detection literature tend to occupy two distinct extremes with respect to training data and model scale. Some studies employ very small datasets of fewer than 100 scans [23,56], limiting statistical power and generalizability. Others rely on most or all of the RSNA training set, often with large backbones or ensemble configurations totaling over 100 million parameters [16,24]. The proposed framework adopts an intermediate strategy, using 6000 stratified RSNA studies and a compact single-backbone architecture—the EfficientNetV2-S backbone contains approximately 22 million parameters, less than a quarter of the size of architectures commonly used for ICH detection, including larger single-CNN models [16,29,42] and ensemble-based approaches [24]—that can be trained on a single GPU in under 26 h. This design does not sacrifice competitive performance: the framework achieves strong internal results and maintains discrimination performance comparable to substantially larger systems on external validation cohorts. The primary motivation for this resource-constrained design is not solely efficiency, but reproducibility—the ability for independent institutions to retrain, audit, and adapt the model without requiring large-scale infrastructure.

A methodologically important distinction concerns the treatment of scan-level prediction. Many prior approaches optimize slice-level classification and derive scan-level outputs through post hoc aggregation, typically via max pooling or related heuristics [35,56]. Others focus exclusively on scan-level outputs without retaining slice-level supervision. The proposed framework instead incorporates a dedicated scan-level prediction pathway with explicit scan-level supervision, trained jointly with the slice-level objective. This design is motivated by clinical relevance: treatment decisions are made at the study level, and scan-level performance is therefore the most directly actionable output for triage workflows. At the same time, slice-level supervision is preserved because it provides substantially denser training signals, improves optimization stability, and encourages the learning of localized hemorrhage representations—a foundation for future systems capable of localization and segmentation. Joint optimization of both objectives therefore offers a practical compromise between clinical relevance and effective representation learning. Reporting both slice- and scan-level performance provides a more complete characterization of model behavior and facilitates comparison across studies emphasizing different evaluation levels.

External validation remains limited across much of the ICH detection literature, with many studies relying exclusively on held-out splits from a single dataset. The proposed framework was evaluated on two independent external cohorts—PhysioNet and CQ500—that introduce geographic variation, demographic differences, and technical distribution shifts in acquisition protocol. The results suggest that robust generalization and competitive performance can be achieved without requiring the substantial computational resources commonly associated with contemporary medical imaging systems.

### 5.5. Limitations and Future Work

Several limitations should be acknowledged alongside directions for future research.

First, the framework does not provide spatial localization of hemorrhages within slices. The sequence module integrates contextual information across slices but produces classification outputs without spatial grounding. Incorporating gradient-based attribution methods such as Grad-CAM, attention visualization, or dedicated segmentation modules could enhance clinical utility by highlighting specific regions of interest for radiologist review, supporting more efficient interpretation workflows and enabling future studies to characterize localization performance.

Second, although external validation was substantially strengthened through the inclusion of both PhysioNet and CQ500, both datasets remain retrospective and the PhysioNet cohort is relatively small. Although the hybrid model consistently showed numerical improvements in discrimination metrics across both external datasets, these AUROC differences did not reach statistical significance. Furthermore, strong performance on CQ500 should not be interpreted as evidence of generalization to any unseen acquisition protocol, but rather to the specific technical shift it represents. Robustness under other untested conditions cannot be assumed. Clinical deployment should therefore be preceded by local validation on the target setting’s acquisition parameters, potentially requiring additional fine-tuning on setting-specific data.

Third, the framework was trained on a stratified subset comprising 28% of the RSNA dataset. While this design was chosen to evaluate resource-efficient training, it may underrepresent rare hemorrhage presentations compared with models trained on the full cohort, and performance on uncommon subtypes should be interpreted with appropriate caution.

Fourth, while the hybrid architecture consistently outperformed the backbone across all datasets, the present study was not designed to isolate the individual contribution of each architectural component. Future work should include dedicated ablation analyses of the sequence module, positional encoding strategy, and multi-pooling aggregation mechanism. Such analyses would clarify the relative contribution of inter-slice modeling, spatial order information, and pooling design to the observed performance gains, providing stronger justification for each architectural decision.

Finally, while this work prioritized computational efficiency and used established CNN and RNN architectures, emerging architectures may offer additional performance gains. Transformer-based models for both slice-level representation learning and scan-level aggregation represent promising directions. Lightweight Transformer variants, including efficient attention mechanisms and hybrid CNN-Transformer architectures, may offer favorable trade-offs between accuracy and computational requirements while maintaining compatibility with resource-constrained settings.

## 6. Conclusions

Intracranial hemorrhage (ICH) is a time-critical neurological emergency for which delays in CT interpretation can adversely affect patient outcomes. Automated detection systems may support radiological triage, particularly in high-volume or resource-constrained clinical environments.

This study presented a resource-efficient hybrid DL framework for automated ICH detection that combines EfficientNetV2-S-based slice-level feature extraction with bidirectional GRU-based sequential modeling for scan-level prediction. On the held-out RSNA test set, the proposed model achieved a scan-level AUROC of 0.980 and an AUPRC of 0.977. The sequence modeling component improved classification performance over the CNN backbone across all datasets and evaluation levels; the corresponding improvement in AUROC reached statistical significance on the internal RSNA test set but not on the external cohorts. The largest gains were observed in threshold-dependent metrics. Across all three datasets, the hybrid architecture reduced false-positive scan assignments and improved F1-score, accuracy, and precision, demonstrating that inter-slice contextual modeling provides practical operational benefits beyond improvements in ranking metrics alone.

External validation on the PhysioNet and CQ500 datasets further demonstrated the model’s ability to generalize across datasets with different acquisition protocols, geographic origins, and patient populations, achieving scan-level AUROCs of 0.914 and 0.905, respectively. These results indicate that competitive performance and consistent operational improvements can be achieved with a compact architecture requiring substantially fewer computational resources than many existing approaches, while maintaining strong absolute performance despite geographic and technical distribution shifts.

Overall, the proposed framework demonstrates that effective ICH detection does not necessarily require large-scale architectures or extensive computational resources. By combining efficient feature extraction, inter-slice contextual modeling, and validation across multiple external cohorts, the framework provides a practical and reproducible approach for automated ICH detection. Future work should focus on localization capabilities, larger prospective multi-center validation studies, and emerging lightweight Transformer-based architectures. 

## Figures and Tables

**Table 1:** Commonly used CT windowing settings in disease detection literature.

Window Type	WC (HU)	WW (HU)	References
Brain	40	80	[14,22,23,24,33,34,35]
Blood/Subdural	80	200	[14,22,24,33,34,35]
Bone	600	2800	[22,23,24,33]
Soft Tissue	20–60	350–400	[34,35]

**Table 2:** Stage-2 training RSNA Dataset distribution.

Label	Slices		Scans	
	Samples	Percentage (%)	Samples	Percentage (%)
None	644,870	85.66	12,862	59.15
ICH (Any)	107,933	14.34	8882	40.85
IPH	36,118	4.80	5321	24.47
IVH	26,205	3.48	3692	16.98
SAH	35,675	4.74	3932	18.08
SDH	47,166	6.27	3812	17.53
EDH	3145	0.42	354	1.63
Total	752,803		21,744	

**Table 3:** Dataset distribution across train, validation, and test sets.

Set	Label	Slices		Scans	
		Samples	Percentage	Samples	Percentage
Train	0	130,324	85.5	2608	59.3
	1	22,060	14.5	1792	40.7
Val.	0	17,698	85.4	355	59.2
	1	3027	14.6	245	40.8
Test	0	29,505	85.6	592	59.2
	1	4978	14.4	408	40.8
Total		207,592		6000	

**Table 4:** Slice thickness distribution across CQ500 studies.

Slice Thickness (mm)	Studies	Percentage (%)
0.625	354	75.0
5.000	42	8.9
3.000	37	7.8
1.000	21	4.4
1.500	13	2.8
1.250	4	0.8
0.750	1	0.2
Total	472	100.0

**Table 5:** CQ500 agreement subset label distribution.

Label	Slices		Scans	
	Samples	Percentage (%)	Samples	Percentage (%)
Negative	79,155	87.5	250	60.1
Positive	11,267	12.5	166	39.9
Total	90,422		416	

**Table 6:** Implemented TrivialAugment-like strategy used.

Transform	Description
Vertical Flip	Flips the image along the vertical axis.
Rotation	Rotates the image by an angle sampled uniformly within the defined rotation range.
Shear X	Applies a horizontal shear with a magnitude sampled uniformly within the shear range.
Shear Y	Applies a vertical shear with a magnitude sampled uniformly within the shear range.
Translate X	Shifts the image horizontally by a distance sampled uniformly within the translation range (relative to image size).
Translate Y	Shifts the image vertically by a distance sampled uniformly within the translation range (relative to image size).
Identity	No transformation is applied; the image remains unchanged.

**Table 7:** Comparison of pretrained CNN architectures on ImageNet-1K. Acc@1 and Acc@5 represent top-1 and top-5 accuracy, respectively. Models are ordered by parameter count.

Model	Acc@1 (%)	Acc@5 (%)	Parameters (M)
ViT-L/16	85.146	97.422	304.3
VGG-16	71.592	90.382	138.4
ResNeXt-101 32x8d	82.834	96.228	88.8
RegNet-Y 8GF	82.828	96.330	39.4
MaxVit-T	83.700	96.722	30.9
DenseNet-161	77.138	93.560	28.7
ConvNeXt-Tiny	82.520	96.146	28.6
Swin-T	81.474	95.776	28.3
ResNet-34	73.314	91.420	21.8
**EfficientNet-V2-S**	**84.228**	**96.878**	**21.5**
EfficientNet-B4	83.384	96.594	19.3
DenseNet-169	75.600	92.806	14.1
DenseNet-121	74.434	91.972	8.0

**Table 8:** Impact of windowing strategy and augmentation on CNN backbone slice-level performance on the RSNA validation and test sets.

Metric	Sigmoid Multi-Window Level 2 Augmentation		Single-Window No Augmentation	
	Test	Validation	Test	Validation
BCE Loss	0.143	0.156	0.387	0.376
AUROC	0.972	0.969	0.800	0.813
AUPRC	0.901	0.887	0.417	0.465
Accuracy	0.950	0.947	0.862	0.867
F1-Score	0.821	0.811	0.294	0.322
Precision	0.855	0.850	0.561	0.624
Recall	0.789	0.776	0.199	0.217

**Table 9:** Impact of dual-level loss weighting on hybrid model scan-level and slice-level performance on the RSNA test set.

Metric	Scan-Level			Slice-Level		
	Equal Weights	Scan Focused	Slice Focused	Equal Weights	Scan Focused	Slice Focused
BCE Loss	0.181	0.187	0.202	0.126	0.130	0.131
AUROC	0.980	0.981	0.979	0.981	0.981	0.981
AUPRC	0.977	0.978	0.976	0.923	0.921	0.923
Accuracy	0.941	0.937	0.936	0.955	0.954	0.954
F1-Score	0.927	0.925	0.922	0.837	0.836	0.837
Precision	0.942	0.939	0.920	0.874	0.866	0.859
Recall	0.912	0.912	0.924	0.802	0.809	0.817

**Table 10:** Final hybrid model scan-level performance metrics on the RSNA test and validation sets, confirming no overfitting at the scan level.

Metric	Test Set	Validation Set
BCE Loss	0.181	0.172
AUROC	0.980	0.983
AUPRC	0.977	0.977
Accuracy	0.941	0.942
F1-Score	0.927	0.927
Precision	0.942	0.945
Recall	0.912	0.910

**Table 11:** Scan-level performance comparison between the CNN backbone and the hybrid model on the RSNA test set.

Metric	Backbone	Hybrid
AUROC	0.977	0.980
AUPRC	0.975	0.977
Accuracy	0.910	0.941
F1-Score	0.896	0.927
Precision	0.846	0.942
Recall	0.953	0.912
TN/FP/FN/TP	521/71/19/389	569/23/36/372
DeLong AUROC SE	±0.0046	±0.0041
DeLong *p*-value (standard)	0.039	

**Table 12:** Slice-level performance comparison between the CNN backbone and the hybrid model on the RSNA test set.

Metric	Backbone	Hybrid
AUROC	0.972	0.981
AUPRC	0.901	0.923
Accuracy	0.950	0.955
F1-Score	0.821	0.837
Precision	0.855	0.874
Recall	0.789	0.802
TN/FP/FN/TP	28,838/667/1051/3927	28,932/573/987/3991
DeLong *p*-value (standard)	$2.90\times 10^{-63}$	
DeLong *p*-value (clustered)	0.241	

**Table 13:** Scan-level performance comparison between the CNN backbone and the hybrid model on the PhysioNet dataset.

Metric	Backbone	Hybrid
AUROC	0.900	0.914
AUPRC	0.921	0.932
Accuracy	0.653	0.827
F1-Score	0.729	0.817
Precision	0.583	0.829
Recall	0.972	0.806
TN/FP/FN/TP	14/25/1/35	33/6/7/29
DeLong AUROC SE	±0.0364	±0.0329
DeLong *p*-value (standard)	0.186	

**Table 14:** Slice-level performance comparison between the CNN backbone and the hybrid model on the PhysioNet dataset.

Metric	Backbone	Hybrid
AUROC	0.951	0.960
AUPRC	0.813	0.836
Accuracy	0.918	0.943
F1-Score	0.678	0.755
Precision	0.611	0.737
Recall	0.761	0.774
TN/FP/FN/TP	2342/154/76/242	2408/88/72/246
DeLong *p*-value (standard)	$7.71\times 10^{-5}$	
DeLong *p*-value (clustered)	0.781	

**Table 15:** Scan-level performance comparison between the CNN backbone and the hybrid model on the CQ500 external validation set (472 studies).

Metric	Backbone	Hybrid
AUROC	0.900	0.905
AUPRC	0.906	0.909
Accuracy	0.621	0.820
F1-Score	0.674	0.799
Precision	0.526	0.748
Recall	0.939	0.858
TN/FP/FN/TP	108/167/12/185	218/57/28/169
DeLong AUROC SE	±0.0159	±0.0152
DeLong *p*-value (standard)	0.339	

**Table 16:** Slice-level performance comparison between the CNN backbone and the hybrid model on the CQ500 agreement subset (416 studies).

Metric	Backbone	Hybrid
AUROC	0.928	0.937
AUPRC	0.758	0.772
Accuracy	0.935	0.938
F1-Score	0.712	0.720
Precision	0.797	0.826
Recall	0.643	0.639
TN/FP/FN/TP	77,312/1843/4021/7246	77,637/1518/4073/7194
DeLong *p*-value (standard)	$4.14\times 10^{-97}$	
DeLong *p*-value (clustered)	0.921	

**Table 17:** Comparison of representative deep learning approaches for intracranial hemorrhage detection.

Study	Backbone	Sequence Model	Training Data	External Validation	Windowing/ Aug. Analysis	Direct Scan- Level Loss	Slice + Scan Metrics
Ye et al. [16]	138 M	RNN	2537/299	×	×	✓	×
Wang et al. [24]	∼110 M	BiGRU + GRU	19,530/2214	✓	×	×	✓
Zhang et al. [35]	N/R	3D Conv	19,530/2214	×	×	×	×
Kumar et al. [23]	N/R	None	82 scans	×	×	×	×
Wang et al. [33]	∼22 M + ViT	None	25,000+ scans	✓	✓	×	×
Malik et al. [56]	12 M	None	82 scans	×	×	×	×
**Proposed Method**	**22 M**	**BiGRU**	**5000/1000**	✓	✓	✓	✓

✓ = feature present/reported; × = feature absent or not reported.

## Data Availability

The data used in this study are publicly available. The RSNA Intracranial Hemorrhage Detection dataset is accessible through the RSNA official challenge page at https://www.rsna.org/artificial-intelligence/ai-image-challenge/rsna-intracranial-hemorrhage-detection-challenge-2019 (accessed on 15 October 2025). The PhysioNet CT Images for Intracranial Hemorrhage Detection and Segmentation dataset is available at https://physionet.org/content/ct-ich/1.3.1/ (accessed on 21 November 2025). The qure.ai CQ500 dataset is available at https://www.kaggle.com/datasets/crawford/qureai-headct (accessed on 13 June 2026). No new data were created in this study.

## References

[1] Freeman W.D., Aguilar M.I. (2012). Intracranial Hemorrhage: Diagnosis and Management. Neurol. Clin..

[2] de Oliveira Manoel A.L., Goffi A., Zampieri F.G., Turkel-Parrella D., Duggal A., Marotta T.R., Macdonald R.L., Abrahamson S. (2016). The critical care management of spontaneous intracranial hemorrhage: A contemporary review. Crit. Care.

[3] Li W., Ruan X., Yang H., Zhang S., Rui F., Xiong J. (2025). Global, regional and national trends in the burden of intracranial hemorrhage, 1990–2021: Results from the Global Burden of Disease study. Heliyon.

[4] Caceres J.A., Goldstein J.N. (2012). Intracranial Hemorrhage. Emerg. Med. Clin. N. Am..

[5] Bohr A., Memarzadeh K. (2020). The rise of artificial intelligence in healthcare applications. Artificial Intelligence in Healthcare.

[6] Thomas K., Edpuganti S., Puthooran D., Thomas A., Joy A., Latheef S. (2025). Artificial intelligence in modern clinical practice. Med. Int..

[7] Jiang F., Jiang Y., Zhi H., Dong Y., Li H., Ma S., Wang Y., Dong Q., Shen H., Wang Y. (2017). Artificial intelligence in healthcare: Past, present and future. Stroke Vasc. Neurol..

[8] Park S.H., Han K., Jang H.Y., Park J.E., Lee J.G., Kim D.W., Choi J. (2023). Methods for Clinical Evaluation of Artificial Intelligence Algorithms for Medical Diagnosis. Radiology.

[9] Hosny A., Parmar C., Quackenbush J., Schwartz L.H., Aerts H.J.W.L. (2018). Artificial intelligence in radiology. Nat. Rev. Cancer.

[10] Yamashita R., Nishio M., Do R.K.G., Togashi K. (2018). Convolutional neural networks: An overview and application in radiology. Insights Imaging.

[11] Esteva A., Kuprel B., Novoa R.A., Ko J., Swetter S.M., Blau H.M., Thrun S. (2017). Dermatologist-level classification of skin cancer with deep neural networks. Nature.

[12] Kermany D.S., Goldbaum M., Cai W., Valentim C.C., Liang H., Baxter S.L., McKeown A., Yang G., Wu X., Yan F. (2018). Identifying Medical Diagnoses and Treatable Diseases by Image-Based Deep Learning. Cell.

[13] Matsoukas S., Scaggiante J., Schuldt B.R., Smith C.J., Chennareddy S., Kalagara R., Majidi S., Bederson J.B., Fifi J.T., Mocco J. (2022). Accuracy of artificial intelligence for the detection of intracranial hemorrhage and chronic cerebral microbleeds: A systematic review and pooled analysis. Radiol. Medica.

[14] Lee H., Yune S., Mansouri M., Kim M., Tajmir S.H., Guerrier C.E., Ebert S.A., Pomerantz S.R., Romero J.M., Kamalian S. (2018). An explainable deep-learning algorithm for the detection of acute intracranial haemorrhage from small datasets. Nat. Biomed. Eng..

[15] Kuo W., Häne C., Mukherjee P., Malik J., Yuh E.L. (2019). Expert-level detection of acute intracranial hemorrhage on head computed tomography using deep learning. Proc. Natl. Acad. Sci. USA.

[16] Ye H., Gao F., Yin Y., Guo D., Zhao P., Lu Y., Wang X., Bai J., Cao K., Song Q. (2019). Precise diagnosis of intracranial hemorrhage and subtypes using a three-dimensional joint convolutional and recurrent neural network. Eur. Radiol..

[17] Monica Jenefer B.M., Senathipathi K., Aarthi, Annapandi (2022). Detection and categorization of acute intracranial hemorrhage subtypes using a multilayer DenseNet-ResNet architecture with improved random forest classifier. Concurr. Comput. Pract. Exp..

[18] Zhao X., Chen K., Wu G., Zhang G., Zhou X., Lv C., Wu S., Chen Y., Xie G., Yao Z. (2021). Deep learning shows good reliability for automatic segmentation and volume measurement of brain hemorrhage, intraventricular extension, and peripheral edema. Eur. Radiol..

[19] Ironside N., Chen C.J., Mutasa S., Sim J.L., Marfatia S., Roh D., Ding D., Mayer S.A., Lignelli A., Connolly E.S. (2019). Fully Automated Segmentation Algorithm for Hematoma Volumetric Analysis in Spontaneous Intracerebral Hemorrhage. Stroke.

[20] Arab A., Chinda B., Medvedev G., Siu W., Guo H., Gu T., Moreno S., Hamarneh G., Ester M., Song X. (2020). A fast and fully-automated deep-learning approach for accurate hemorrhage segmentation and volume quantification in non-contrast whole-head CT. Sci. Rep..

[21] Ahmed S.N., Prakasam P. (2025). Intracranial hemorrhage segmentation and classification framework in computer tomography images using deep learning techniques. Sci. Rep..

[22] Chilamkurthy S., Ghosh R., Tanamala S., Biviji M., Campeau N.G., Venugopal V.K., Mahajan V., Rao P., Warier P. (2018). Deep learning algorithms for detection of critical findings in head CT scans: A retrospective study. Lancet.

[23] Kumar Kar N., Jana S., Rahman A., Rahul Ashokrao P.G.I., Alarmelu Mangai R. Automated Intracranial Hemorrhage Detection Using Deep Learning in Medical Image Analysis. Proceedings of the 2024 International Conference on Data Science and Network Security (ICDSNS).

[24] Wang X., Shen T., Yang S., Lan J., Xu Y., Wang M., Zhang J., Han X. (2021). A deep learning algorithm for automatic detection and classification of acute intracranial hemorrhages in head CT scans. NeuroImage Clin..

[25] Faiz M.N., Badriyah T., Kusuma S.F. Classification of Intracranial Hemorrhage Based on CT-Scan Image with Vision Transformer (ViT) Method. Proceedings of the 2024 International Electronics Symposium (IES).

[26] Rasoulian A., Salari S., Xiao Y. (2022). Weakly Supervised Intracranial Hemorrhage Segmentation Using Hierarchical Combination of Attention Maps from a Swin Transformer. Machine Learning in Clinical Neuroimaging.

[27] Moassefi M., Rouzrokh P., Conte G.M., Vahdati S., Fu T., Tahmasebi A., Younis M., Farahani K., Gentili A., Kline T. (2023). Reproducibility of Deep Learning Algorithms Developed for Medical Imaging Analysis: A Systematic Review. J. Digit. Imaging.

[28] Beam A.L., Manrai A.K., Ghassemi M. (2020). Challenges to the Reproducibility of Machine Learning Models in Health Care. JAMA.

[29] Lezzar F., Mili S.E. (2025). Advanced Deep Learning for Stroke Classification Using Multi-Slice CT Image Analysis. J. Electron. Electromed. Eng. Med. Inform..

[30] Obuchowicz R., Piórkowski A., Nurzyńska K., Obuchowicz B., Strzelecki M., Bielecka M. (2026). Will AI Replace Physicians in the Near Future? AI Adoption Barriers in Medicine. Diagnostics.

[31] Mukherjee J., Kar M., Chakrabarti A., Das S. (2024). Basic terminologies of computed tomography scan. Application of Artificial Intelligence in Early Detection of Lung Cancer.

[32] Yeo M., Tahayori B., Kok H.K., Maingard J., Kutaiba N., Russell J., Thijs V., Jhamb A., Chandra R.V., Brooks M. (2021). Review of deep learning algorithms for the automatic detection of intracranial hemorrhages on computed tomography head imaging. J. Neurointerv. Surg..

[33] Wang X., Liu Z., Li J., Xiong G. Vision Transformer-based Classification Study of Intracranial Hemorrhage. Proceedings of the 2022 3rd International Conference on Computer Vision, Image and Deep Learning & International Conference on Computer Engineering and Applications (CVIDL & ICCEA).

[34] Wu Y., Schmidt A., Hernández-Sánchez E., Molina R., Katsaggelos A.K. (2021). Combining Attention-Based Multiple Instance Learning and Gaussian Processes for CT Hemorrhage Detection. Medical Image Computing and Computer Assisted Intervention–MICCAI 2021.

[35] Zhang L., Miao W., Zhu C., Wang Y., Luo Y., Song R., Liu L., Yang J. (2023). A Weakly Supervised-Guided Soft Attention Network for Classification of Intracranial Hemorrhage. IEEE Trans. Cogn. Dev. Syst..

[36] Lee H., Kim M., Do S. (2018). Practical Window Setting Optimization for Medical Image Deep Learning. arXiv.

[37] Remedios S.W., Wu Z., Bermudez C., Kerley C.I., Roy S., Patel M.B., Butman J.A., Landman B.A., Pham D.L., Landman B.A., Išgum I. (2020). Extracting 2D weak labels from volume labels using multiple instance learning in CT hemorrhage detection. Proceedings of the Medical Imaging 2020: Image Processing.

[38] Huang G., Liu Z., van der Maaten L., Weinberger K.Q. Densely Connected Convolutional Networks. Proceedings of the IEEE Conference on Computer Vision and Pattern Recognition.

[39] He K., Zhang X., Ren S., Sun J. Deep Residual Learning for Image Recognition. Proceedings of the IEEE Conference on Computer Vision and Pattern Recognition.

[40] Rajagopal M., Buradagunta S., Almeshari M., Alzamil Y., Ramalingam R., Ravi V. (2023). An Efficient Framework to Detect Intracranial Hemorrhage Using Hybrid Deep Neural Networks. Brain Sci..

[41] Sreelakshmi D., Inthiyaz S., Prasad L.V.N., Hassan A.R., Hussein M.J., Abbas N. (2023). Identification and prediction of acute intracranial hemorrhage by using CNN and RNN techniques. Proceedings of the Physical Mesomechanics of Condensed Matter: Physical Principles of Multiscale Structure Formation and the Mechanisms of Nonlinear Behavior: MESO2022.

[42] Hossain M.M., Ahmed M.M., Nafi A.A.N., Islam M.R., Ali M.S., Haque J., Miah M.S., Rahman M.M., Islam M.K. (2025). A novel hybrid ViT-LSTM model with explainable AI for brain stroke detection and classification in CT images: A case study of Rajshahi region. Comput. Biol. Med..

[43] Simonyan K., Zisserman A. (2014). Very Deep Convolutional Networks for Large-Scale Image Recognition. arXiv.

[44] Ker J., Singh S.P., Bai Y., Rao J., Lim T., Wang L. (2019). Image Thresholding Improves 3-Dimensional Convolutional Neural Network Diagnosis of Different Acute Brain Hemorrhages on Computed Tomography Scans. Sensors.

[45] Flanders A.E., Prevedello L.M., Shih G., Halabi S.S., Kalpathy-Cramer J., Ball R., Mongan J.T., Stein A., Kitamura F.C., Lungren M.P. (2020). Construction of a Machine Learning Dataset through Collaboration: The RSNA 2019 Brain CT Hemorrhage Challenge. Radiol. Artif. Intell..

[46] Bidgood W.D., Horii S.C., Prior F.W., Van Syckle D.E. (1997). Understanding and Using DICOM, the Data Interchange Standard for Biomedical Imaging. J. Am. Med. Inform. Assoc..

[47] Hssayeni M. (2020). Computed Tomography Images for Intracranial Hemorrhage Detection and Segmentation (Version 1.3.1). PhysioNet. https://physionet.org/content/ct-ich/1.3.1/.

[48] Hssayeni M.D., Croock M.S., Salman A.D., Al-khafaji H.F., Yahya Z.A., Ghoraani B. (2020). Intracranial Hemorrhage Segmentation Using a Deep Convolutional Model. Data.

[49] Reis E.P., Nascimento F., Aranha M., Secol F.M., Machado B., Felix M., Stein A., Amaro E. (2020). Brain Hemorrhage Extended (BHX): Bounding box extrapolation from thick to thin slice CT images. PhysioNet. https://physionet.org/content/bhx-brain-bounding-box/1.1/.

[50] Moore J., Kunis S. (2023). Zarr: A Cloud-Optimized Storage for Interactive Access of Large Arrays. Proc. Conf. Res. Data Infrastruct..

[51] Garcea F., Serra A., Lamberti F., Morra L. (2023). Data augmentation for medical imaging: A systematic literature review. Comput. Biol. Med..

[52] Müller S.G., Hutter F. TrivialAugment: Tuning-free Yet State-of-the-Art Data Augmentation. Proceedings of the IEEE/CVF International Conference on Computer Vision.

[53] Vryniotis V. (2021). How to Train State-Of-The-Art Models Using TorchVision’s Latest Primitives. https://pytorch.org/blog/how-to-train-state-of-the-art-models-using-torchvision-latest-primitives/.

[54] (2025). PyTorch Contributors. Models and Pre-Trained Weights. https://docs.pytorch.org/vision/main/models.html.

[55] (2016). maintainers, T.; Contributors. TorchVision: PyTorch’s Computer Vision Library. https://github.com/pytorch/vision.

[56] Malik P., Dureja A., Dureja A., Rathore R.S., Malhotra N. (2024). Enhancing Intracranial Hemorrhage Diagnosis through Deep Learning Models. Procedia Comput. Sci..

[57] Tan M., Le Q.V. (2021). EfficientNetV2: Smaller Models and Faster Training. arXiv.

[58] Sandler M., Howard A.G., Zhu M., Zhmoginov A., Chen L. (2018). Inverted Residuals and Linear Bottlenecks: Mobile Networks for Classification, Detection and Segmentation. arXiv.

[59] Kingma D.P., Ba J. (2014). Adam: A Method for Stochastic Optimization. arXiv.

[60] Micikevicius P., Narang S., Alben J., Diamos G., Elsen E., Garcia D., Ginsburg B., Houston M., Kuchaiev O., Venkatesh G. (2017). Mixed Precision Training. arXiv.

[61] DeLong E.R., DeLong D.M., Clarke-Pearson D.L. (1988). Comparing the Areas under Two or More Correlated Receiver Operating Characteristic Curves: A Nonparametric Approach. Biometrics.

[62] Sun X., Xu W. (2014). Fast Implementation of DeLong’s Algorithm for Comparing the Areas Under Correlated Receiver Operating Characteristic Curves. IEEE Signal Process. Lett..

